# Imine–Thiocarbamate Hybrid Pincer Systems: From Mechanochemical Activation to Cytotoxicity Evaluation of the Cyclopalladated Derivatives

**DOI:** 10.3390/molecules31030546

**Published:** 2026-02-04

**Authors:** Aleksandr A. Spiridonov, Diana V. Aleksanyan, Dmitry V. Yakshin, Yulia V. Nelyubina, Ekaterina Yu. Rybalkina, Zinaida S. Klemenkova, Vladimir A. Kozlov

**Affiliations:** 1 A. N. Nesmeyanov Institute of Organoelement Compounds, Russian Academy of Sciences, ul. Vavilova 28, str. 1, 119334 Moscow, Russiadmitr.yak2806@yandex.ru (D.V.Y.); zklem@ineos.ac.ru (Z.S.K.); fos@ineos.ac.ru (V.A.K.); 2 Federal Research Center of Problems of Chemical Physics and Medicinal Chemistry, Russian Academy of Sciences, pr. Akademika Semenova 1, 142432 Chernogolovka, Moscow Oblast, Russia; unelya@gmail.com; 3 N. N. Blokhin National Medical Research Center of Oncology of the Ministry of Health of the Russian Federation, Kashirskoe shosse 23, 115478 Moscow, Russia; kate_rybalkina@mail.ru

**Keywords:** pincer complexes, tridentate ligands, palladium, cyclometalation, mechanochemistry, cytotoxicity

## Abstract

Organometallic and metal–organic compounds are widely used in different fields of chemistry and allied disciplines, including bioinorganic and medicinal chemistry. Of particular interest is the development of novel potential anticancer agents based on palladium(II) complexes of the so-called pincer-type ligands, featuring a specific monoanionic tridentate framework. In this work, hybrid imine–thiocarbamate ligands are shown to readily undergo direct cyclopalladation in solution and under solvent-free conditions, in particular upon mechanochemical activation, yielding a series of Pd(II) pincer complexes. The latter exhibit promising cytotoxic activity against several solid and hematopoietic cancer cell lines.

## 1. Introduction

Despite their half-century history [1], pincer-type complexes are not only still actively explored in their traditional fields of application, such as organometallic chemistry and catalysis [2,3,4,5,6,7,8], but also expanding into new spheres, including chemosensing/recognition, supramolecular coordination, bioinorganic and medicinal chemistry [9,10,11,12,13]. Their specific monoanionic tridentate ligand framework offers high stability and tunability, which distinguishes pincer complexes from other popular types of organometallic compounds [13,14,15,16,17,18]. In the design of new potential palladium-based drugs, a pincer type coordination is particularly attractive since it can provide the desired balance between thermodynamic stability and kinetic lability to cope with rapid ligand exchange processes, which comprise the key disadvantage of Pd(II) species compared to their platinum(II) analogs [19]. At the same time, a combination of pendant donor groups of different nature offers an opportunity to achieve a hemilabile coordination of metal ions, which is especially interesting in terms of the diversified reactivity of ensuing complexes in biological media.

**Figure 1:**
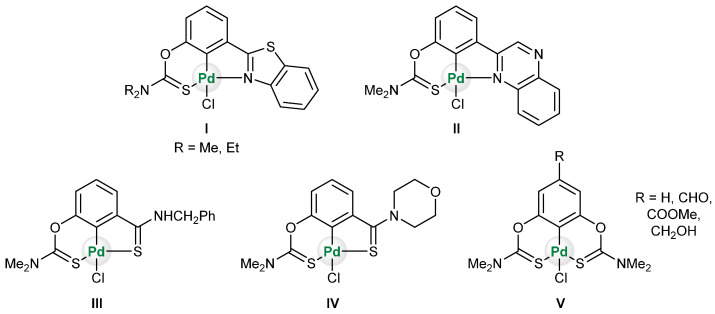
Palladium(II) pincer complexes bearing thiocarbamate ancillary donor groups.

Recently, we have shown that hybrid Pd(II) pincer complexes featuring *S*-donor thiocarbamate and *N*-donor heterocyclic units (benzothiazole or quinoxaline, see Figure 1) exhibit promising antiproliferative activity against several cancer cell lines, affording comparable or even higher efficiency than the clinically used antineoplastic medication cisplatin [20,21]. Furthermore, complexes **I** and **II** were shown to be readily formed under solvent-free conditions upon thermally induced cyclopalladation [20,21]. In continuation of these studies, it seemed interesting to turn to the acyclic imine-based analogs bearing a sterically and electronically disengaged C=N moiety to probe the solid-phase reactivity of new hybrid ligands for developing an ecologically friendly cyclometalation protocol and to evaluate the effect of this structural modification on the cytotoxic activity of resulting Pd(II) pincer complexes.

## 2. Results and Discussion

**Scheme 1:**
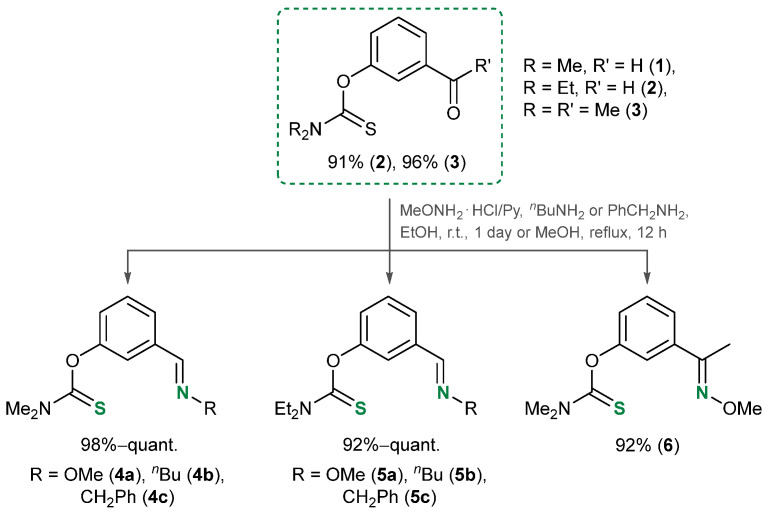
Synthesis of the imine–thiocarbamate hybrid pincer ligands.

A series of imine–thiocarbamate hybrid pincer ligands were readily obtained by the reactions of thiocarbamoyloxy-functionalized carbonyl derivatives **1**–**3** with different amines (Scheme 1). The condensation was smoothly accomplished in ethanol at room temperature, except for that with the acetophenone precursor which required prolonged heating. Imines **4b**,**c** and **5b**,**c** were obtained as mixtures containing a small amount of the initial aldehyde (<6%). Hydrolytically stable oxime derivatives **4a**, **5a**, and **6** were isolated as individual compounds in high yields after purification by column chromatography on silica gel. In turn, the key thiocarbamoyloxy-functionalized precursors were readily obtained upon sequential treatment of *meta*-hydroxy-substituted benzaldehyde or acetophenone with KOH and the corresponding dialkylthiocarbamoyl chloride according to the slightly modified literature procedure (see Section 3) [22,23].

It should be noted that, recently, dimethylthiocarbamoyloxy-substituted benzaldehyde **1** has already been used as a convenient precursor for unsymmetrical thioamide–thiocarbamate pincer ligands, which were found to readily undergo direct cyclopalladation both in solution and under solvent-free conditions, affording Pd(II) complexes **III** and **IV** (Figure 1) in high yields [24]. More importantly, the solid-phase synthesis of these palladacycles as well as their symmetrical bis(thiocarbamate) counterparts **V** (Figure 1), unlike hybrid *S*,*C*,*N*-derivatives **I** and **II**, did not require additional thermal treatment and smoothly proceeded upon mechanochemical activation [24]. However, a difference in the reactivity of bis(thione) and mixed-donor ligands towards Pd(II) ions is not surprising. The detailed research on various systems, summarized in the early fundamental literature on pincer chemistry [25], underscored less favorable preliminary coordination of *N*-donor ligands compared to their *P*- and *S*-containing counterparts. Furthermore, the cyclometalation of *N*-donor ligands can be realized through the kinetically controlled pathway rather than the thermodynamically preferred route, leading to the activation of different ligand sites. Therefore, our goal was to modify the *N*-donor pendant arm in a hybrid imine–thiocarbamate pincer system so that to facilitate its cyclopalladation in solution and in the solid state.

**Scheme 2:**
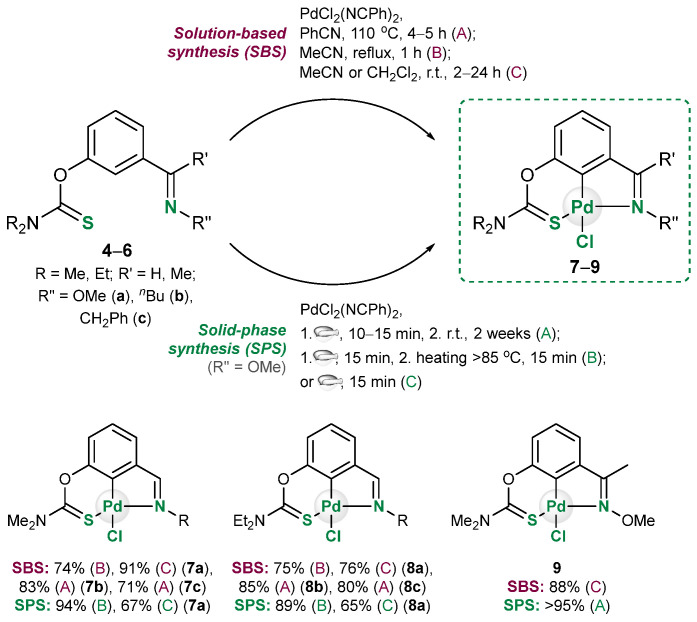
Cyclopalladation of the imine–thiocarbamate hybrid pincer ligands in solution and under solvent-free conditions.

The cyclometalation of imine–thiocarbamate ligands **4b**,**c** and **5b**,**c** was accomplished upon heating with PdCl_2_(NCPh)_2_ in benzonitrile at ca. 110 °C for several hours (Scheme 2, solution-based synthesis (SBS) method A). Their oxime-based analogs **4a** and **5a** were shown to undergo direct cyclopalladation under the action of the same metalating agent already during short-term refluxing in MeCN (SBS method B). Alternatively, palladacycles **7a** and **8a** can be obtained in dichloromethane at room temperature (SBS method C). Therewith, essential discoloration of the reaction mixture with dimethylthiocarbamate derivative **4a** was observed already during the first two hours, while the reaction mixture with its diethylamino-substituted analog **5a** still remained intensely colored, even after 1 day. Furthermore, the chromatographic purification, especially in the case of ligand **5a**, was accompanied by the appearance of a relatively large dark band on the column, indicative of some decomposition processes. This may imply the possible formation of the target pincer complex directly on silica gel, where the reaction in CH_2_Cl_2_ under mild conditions actually facilitates only the preliminary coordination of Pd(II) ions. However, in the case of acetophenone-derived ligand **6**, the complete cyclopalladation under the action of PdCl_2_(NCPh)_2_ was achieved in MeCN at room temperature for 30 min, and palladacycle **9** was isolated in the pure form with high yield after simple evaporation of the solvent and rinsing with Et_2_O to remove residual benzonitrile.

**Figure 2:**
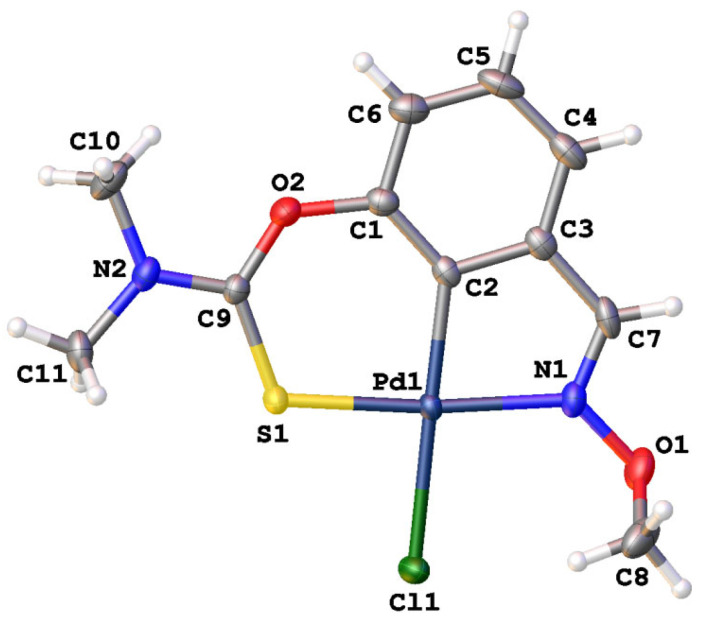

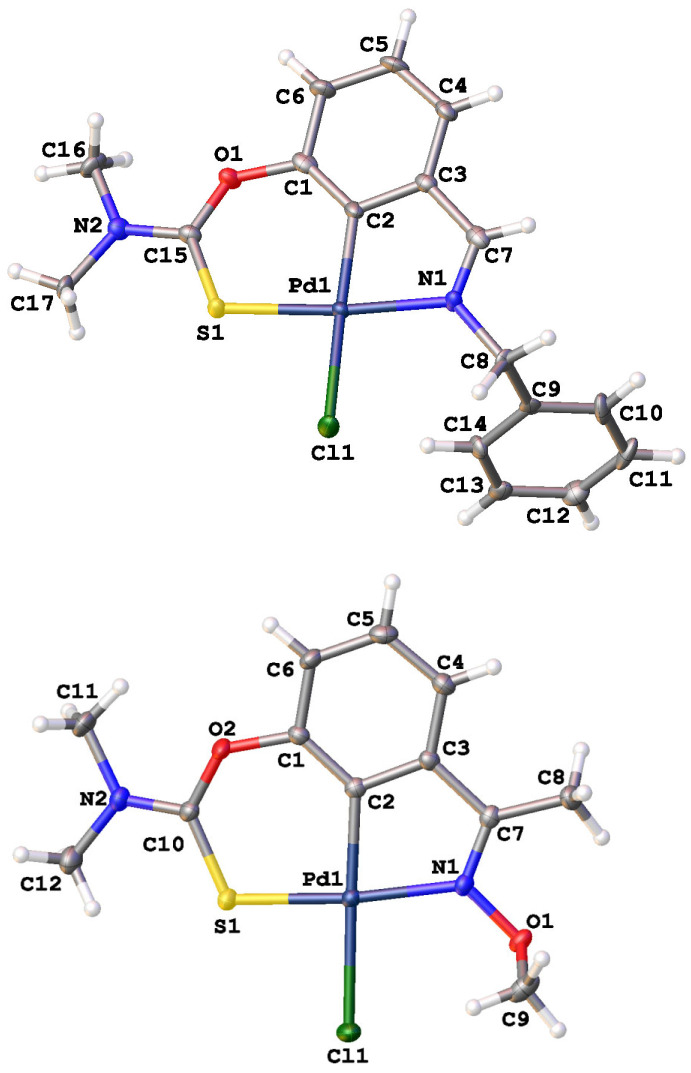
General view of Pd(II) complexes **7a** (**top**), **7c** (**center**), and **9** (**bottom**) in representation of non-hydrogen atoms as thermal ellipsoids at 30% probability level.

A tridentate coordination of the deprotonated imine–thiocarbamate ligands in complexes **7**–**9** was unequivocally confirmed by NMR and IR spectroscopy. The characteristic spectral features included a reduction in the total integral intensity in the field of aromatic proton signals by 1H with concomitant change in the position (Δ*δ*_C_ > 12.1 ppm) and polarity of C2 carbon resonance as a result of metalation. Binding of the ancillary donor groups was evidenced, in particular, by the shifts in the corresponding absorption bands, associated with the imine (Δ*ν* up to 37 cm^−1^) and thiocarbamate (Δ*ν* up to 22 cm^−1^) moieties. For the NMR and IR spectra of the selected ligand/complex pairs, see Figures S1–S18 in the Supporting Information. The identities of the resulting palladacycles were supported by elemental analyses. Finally, X-ray diffraction studies unambiguously confirmed the realization of pincer-type coordination in complexes **7a**, **7c** and **9** (Figure 2). In all cases, the metal ions adopted slightly distorted square-planar geometry, with the main bond distances varying in the expected ranges: Pd–C 1.967–1.977 Å, Pd–Cl 2.377–2.394 Å, Pd–N 2.039–2.055 Å, and Pd–S 2.228–2.247 Å.

To investigate the solid-phase reactivity, compounds **4b**,**c** were chosen as representative examples of the imine-based derivatives. Both ligands were found to form the target Pd(II) pincer complexes upon heating of the ground mixtures with PdCl_2_(NCPh)_2_ without addition of a solvent. However, even the first step (grinding of the reactants) was accompanied by partial hydrolysis of the C=N bond, which intensified during further heating (see, for example, Figures S19–S22 in the Supporting Information). At the same time, their oxime analogs **4a** and **5a** smoothly underwent thermally induced solid-phase cyclopalladation, affording the desired Pd(II) pincer complexes in yields comparable to those observed in the solution-based experiments (compare SBS methods B, C with the solid-phase synthesis (SPS) method B in Scheme 2; for the corresponding IR spectra, see, for example, Figures S23–S26 in the Supporting Information). Although the pure products can be isolated only after purification by column chromatography, the solid-phase methodology is advantageous over the conventional synthesis in solution owing to a significant rate enhancement. Moreover, the target oxime-based complexes were effectively produced after the chromatographic purification of solid residues obtained by simple grinding of the reactants in a mortar, avoiding the heating step (Scheme 2, SPS method C). This once again indicates the possibility of promoting cyclopalladation by silica gel. It should be emphasized that the described reactions are the first examples of solid-phase cyclometalation of liquid pincer-type ligands.

**Figure 3:**
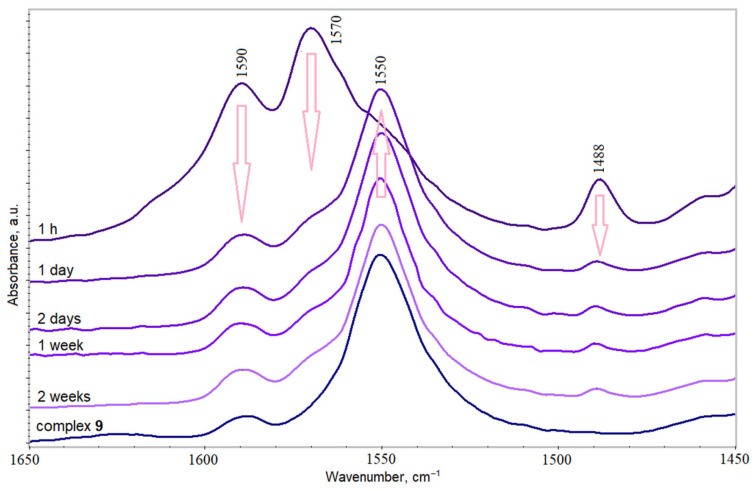
Monitoring the solid-phase cyclopalladation of ligand **6** by IR spectroscopy (the spectra of the solid residue, obtained after grinding the reactants in a mortar and kept at room temperature in a closed glass tube, were registered in the mentioned time periods after grinding).

To our delight, the cyclometalation of acetophenone-derived oxime **6** was initiated by grinding of the reactants in a mortar and smoothly proceeded at room temperature, without the need for additional energy supply (Scheme 2). Monitoring by IR spectroscopy revealed the formation of a coordination predecessor during the mechanochemical activation (with the markedly shifted thiocarbamate absorption band (from 1540 to 1570 cm^−1^) and almost unchanged region of out-of-plane aromatic CH bending vibrations (ca. 680–870 cm^−1^) compared to the spectrum of free ligand **6**) and its slow conversion to the pincer-type product in the solid state (see Figure 3 and Figures S15, S18, S27–S31 in the Supporting Information). Almost complete transformation was observed in 2 weeks. The ^1^H NMR spectrum of the resulting free-flowing powder, registered directly after dissolution in CDCl_3_, confirmed the formation of about 95% of the target pincer product (Figure S32 in the Supporting Information). In our opinion, the cyclopalladation under conditions of such an aging process is likely to be caused by slow dissolution of the coordination precursor in a small amount of benzonitrile, which was released from the metalating agent and did not evaporate during grinding. The realization of LAG (liquid-assisted grinding) conditions was earlier demonstrated to serve as a reason for the effective cyclopalladation in related thiocarbamate pincer systems [24]. The relaxation nature of this long-term process (e.g., crystallization of an amorphous phase) also cannot be excluded.

The resulting pincer complexes were tested for cytotoxic activity against several human solid and hematopoietic cancer cell lines. The conventional cell viability assay involving the MTT dye afforded the half-maximal inhibitory concentrations (exposure time: 48 h); the corresponding data are summarized in Table 1 and Table 2. Human embryonic kidney (HEK293) and breast epithelial (HBL100) cells were used to estimate potential selectivity of the palladacycles under consideration. Cisplatin was employed as a positive control.

As can be seen, imine-based derivatives **7b**,**c** and **8b**,**c** (entries 2, 3 and 5, 6 in Table 1 and Table 2) exhibited a generally high level of activity, markedly outperforming the clinically used platinum-based drug. Therewith, ligand **5b**, chosen as a representative example of the free imine–thiocarbamate hybrids, appeared to be nontoxic at concentrations as high as 30 μM (in the case of the hematopoietic cell lines explored) and 60 μM (in the case of the solid cancer cells), indicating that the cytotoxic effects of the cyclometallated derivatives are stipulated by the coordination with Pd(II) ions. Among the solid cancer lines explored, prostate cancer cells PC3 demonstrated the greatest sensitivity. Even higher efficiency was observed on the hematopoietic cell cultures, for which the values of IC_50_ fell into the low micromolar range, providing the selectivity indices over non-cancerous cells HEK293 up to 5.9. Comparable or better results were achieved on pseudonormal cells HBL100. Of particular importance are similar cytotoxic effects of complexes **7**–**9** on chronic myelogenous leukemia cells K562 and their doxorubicin-resistant clones K562/iS9, which implies the potential of overcoming drug resistance. Oxime-based derivatives **7a**, **8a**, and **9** (entries 1, 4 and 9 in Table 1 and Table 2) appeared to be less effective than their counterparts bearing *n*-butyl and benzyl substituents towards the solid cancer cell lines, but displayed an appreciable level of efficiency on the hematopoietic cell lineages. In general, the imine–thiocarbamate hybrid complexes obtained in this work, although seem to be inferior to their heterocyclic analogs **I** and **II** in terms of activity against solid cancer cell lines, are commensurately effective against the hematopoietic cell lineages and significantly surpass in the anticancer potential some earlier reported Pd(II) pincer complexes [26,27,28]. The possibility of solid-phase synthesis of the imine-based complexes under conditions of mechanochemical activation makes them attractive objects for further examination.

## 3. Experimental Section

### 3.1. General Remarks

All manipulations were carried out in the normal atmosphere without taking precautions to exclude air. Dichloromethane and acetonitrile were distilled from P_2_O_5_. Tetrahydrofuran was distilled over sodium. Carbonyl precursor **1** was obtained from 3-hydroxybenzaldehyde upon sequential treatment with KOH and ClC(S)NMe_2_ according to the published procedure [22,23]. Diethylthiocarbamoyl chloride was prepared from tetraethylthiuram disulfide upon treatment with sulfuryl chloride [29]. All other chemicals and solvents were used as purchased.

The NMR spectra were recorded on Bruker Avance 300 and Avance 400 spectrometers (Bruker AXS GmbH, Karlsruhe, Germany). The chemical shifts (*δ*) were referenced internally by the residual (^1^H) or deuterated (^13^C) solvent signals relative to tetramethylsilane. In all cases, the ^13^C{^1^H} NMR spectra were registered using the *J*MODECHO mode; the signals for the C nuclei bearing odd and even numbers of protons had opposite polarities. For the NMR spectra of the representative ligands and their cyclopalladated derivatives, see Figures S1–S18 in the Supporting Information.

The IR spectra were recorded on a Nicolet Magna-IR750 FT spectrometer (Nicolet, Madison, WI, USA) (resolution 2 cm^−1^, 128 scans). The assignment of absorption bands in the IR spectra was made according to Ref. [30].

Column chromatography was carried out using Macherey-Nagel silica gel 60 (MN Kieselgel 60, 70–230 mesh) (Macherey-Nagel, Dueren, Germany).

The melting points were determined with an MPA 120 EZ-Melt automated melting point apparatus (Stanford Research Systems, Sunnyvale, CA, USA).

### 3.2. Syntheses

#### 3.2.1. Synthesis of Key Carbonyl Precursors **2**, **3**

***O*-(3-Formylphenyl) diethylthiocarbamate 2**


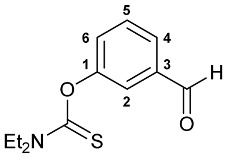


Potassium hydroxide (1.230 g, 21.923 mmol) was added to a suspension of 3-hydroxybenzaldehyde (2.080 g, 17.032 mmol) in distilled water (20 mL) at ca. 5 °C. The resulting mixture was stirred for 15 min. Then a solution of *N*,*N*-diethylthiocarbamoyl chloride (2.583 g, 17.032 mmol) in THF (10 mL) was added dropwise at 0–5 °C. The reaction mixture was stirred upon cooling for 2 h and poured onto ice. The resulting mixture was transferred to a separating funnel, and the target product was extracted with dichloromethane. The aqueous phase was additionally extracted with CH_2_Cl_2_. The combined organic phase was dried over anhydrous Na_2_SO_4_ and evaporated to dryness. The residue obtained was purified by column chromatography on silica gel (eluent: first CH_2_Cl_2_–hexane (1:1) (to separate an admixture arising from the decomposition of Et_2_NC(S)Cl), then neat CH_2_Cl_2_) to give 3.676 g of compound **2** as a light-yellow viscous oil. Yield: 91%. ^1^H NMR (300.13 MHz, CDCl_3_): *δ* 1.32–1.38 (m, 6H, Me in NEt_2_), 3.73 and 3.92 (both q, 2H + 2H, CH_2_ in NEt_2_, ^3^*J*_HH_ = 7.1 Hz), 7.35–7.39 (m, 1H, H_Ar_), 7.55–7.61 (m, 2H, H_Ar_), 7.79 (ddd, 1H, H(C6) or H(C4), ^3^*J*_HH_ = 7.7 Hz, ^4^*J*_HH_ = 1.4 Hz), 10.03 (s, 1H, CHO) ppm. ^13^C{^1^H} NMR (100.61 MHz, CDCl_3_): *δ* 11.78 and 13.60 (both s, Me in NEt_2_), 44.46 and 48.56 (both s, CH_2_ in NEt_2_), 123.48, 127.37, 129.23 and 129.80 (four s, C2, C4–C6), 137.52 (s, C3), 154.47 (s, C1), 186.24 (s, C=S), 191.31 (s, CHO) ppm. IR (thin film, *v*/cm^−1^): 648(w), 693(w), 735(s), 762(w), 777(w), 795(w), 888(w), 913(w), 961(vw), 973(vw), 1001(vw), 1079(w), 1096(w), 1114(m), 1142(m), 1165(m), 1219(s), 1252(m), 1271(m), 1286(m), 1316(m), 1349(w), 1361(w), 1382(m), 1432(m), 1445(m), 1481(m), 1517(br, s) (OC(S)N), 1589(m), 1604(vw), 1702(s) (*ν*C=O), 2733(vw), 2837(vw), 2875(vw), 2936(w), 2980(w), 3056(vw). Anal. Calcd for C_12_H_15_NO_2_S: C, 60.73; H, 6.37; N, 5.90. Found: C, 60.34; H, 6.34; N, 6.21%.

***O*-(3-Acetylphenyl) dimethylthiocarbamate 3**


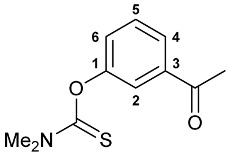


Potassium hydroxide (1.359 g, 24.222 mmol) was added to a suspension of 3-hydroxyacetophenone (3.000 g, 22.035 mmol) in distilled water (25 mL) at ca. 5 °C. The resulting mixture was stirred for 20 min. Then a solution of *N*,*N*-dimethylthiocarbamoyl chloride (2.724 g, 22.039 mmol) in THF (20 mL) was added dropwise at 0–5 °C. The reaction mixture was stirred upon cooling for 2 h and poured onto ice. The resulting mixture was transferred to a separating funnel, and the target product was extracted with dichloromethane. The aqueous phase was additionally extracted with CH_2_Cl_2_. The combined organic phase was dried over anhydrous Na_2_SO_4_ and evaporated to dryness. The resulting oily residue slowly crystallized and, after recrystallization from hexane, afforded 4.700 g of compound **3** as a white solid. Yield: 96%. Mp: 71–72 °C (hexane). ^1^H NMR (400.13 MHz, CDCl_3_): *δ* 2.62 (s, 3H, Me), 3.38 and 3.48 (both s, 3H + 3H, NMe_2_), 7.31 (dd, 1H, H(C4) or H(C6), ^3^*J*_HH_ = 8.2 Hz, ^4^*J*_HH_ = 2.4 Hz), 7.49–7.53 (m, 1H, H(C5)), 7.66 (br. s, 1H, H(C2)), 7.86 (d, 1H, H(C6) or H(C4), ^3^*J*_HH_ = 7.7 Hz) ppm. ^13^C{^1^H} NMR (100.61 MHz, CDCl_3_): *δ* 26.76 (s, Me), 38.85 and 43.38 (both s, NMe_2_), 122.65, 125.89, 127.82 and 129.33 (four s, C2, C4–C6), 138.27 (s, C3), 154.22 (s, C1), 187.40 (s, C=S), 197.07 (s, C=O) ppm. IR (KBr, *v*/cm^−1^): 588(w), 684(w), 702(w), 725(vw), 804(w), 871(w), 896(w), 948(w), 1000(w), 1023(vw), 1090(w), 1130(m), 1158(w), 1198(m), 1258(s), 1276(m), 1289(m), 1360(m), 1394(m), 1406(m), 1426(w), 1442(m), 1483(w), 1549(br, s) (OC(S)N), 1586(w), 1689(s) (*ν*C=O), 2941(vw), 3066(vw). Anal. Calcd for C_11_H_13_NO_2_S: C, 59.17; H, 5.87; N, 6.27. Found: C, 59.24; H, 5.84; N, 6.38%.

#### 3.2.2. Synthesis of Ligands **4**–**6**

**Method A.** *O*-Methylhydroxylamine hydrochloride (1.2 equiv.) and pyridine (3.7–3.9 equiv.) were added to a solution of the corresponding aldehyde (1.0 equiv.) in EtOH (10 mL). The reaction mixture was stirred at room temperature for 4 h and left under ambient conditions overnight. The resulting solution was evaporated to dryness, and the residue obtained was purified by column chromatography on silica gel (eluent: hexane–EtOAc (5:1)) to give the target ligands as light-yellow viscous oils.

**Method B.** A solution of the corresponding amine (1.0 equiv.) in ethanol (3 mL) was added to a solution of the corresponding aldehyde (1 equiv.) in EtOH (3 mL). The reaction mixture was stirred at room temperature for 2–5 h and left under ambient conditions overnight. The resulting solution was evaporated to dryness to give the target ligands as a light-yellow solid (in the case of **5c**) or viscous oils (in the other cases) in quantitative yields. The products contained <6% admixture of the starting aldehyde (according to the ^1^H NMR spectroscopic data) and were used in the synthesis of target Pd(II) pincer complexes without purification.

**Method C.** A solution of ketone **3** (1.0 equiv.), *O*-methylhydroxylamine hydrochloride (1.2 equiv.), and pyridine (4.0 equiv.) in MeOH (10 mL) was refluxed for 12 h. After cooling to room temperature, the resulting solution was evaporated to dryness, and the residue obtained was purified by column chromatography on silica gel (eluent: hexane–CH_2_Cl_2_ (1:1)) to give the target ligand as a white solid.

***O*-{3-[(Methoxyimino)methyl]phenyl} dimethylthiocarbamate 4a**


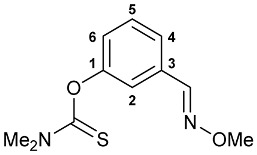


The compound was obtained by **method A** from aldehyde **1** (0.273 g, 1.305 mmol), *O*-methylhydroxylamine hydrochloride (0.130 g, 1.557 mmol), and pyridine (0.41 mL, 5.089 mmol). Yield: 0.303 g (98%). ^1^H NMR (300.13 MHz, CDCl_3_): *δ* 3.36 and 3.47 (both s, 3H + 3H, NMe_2_), 3.98 (s, 3H, OMe), 7.10 (ddd, 1H, H(C4) or H(C6),^3^*J*_HH_ = 7.6 Hz, ^4^*J*_HH_ = 2.1 Hz), 7.35 (br. s, 1H, H(C2)), 7.38–7.47 (m, 2H, H(C5) + H(C6) or H(C4)), 8.06 (s, 1H, CH=N) ppm. ^13^C{^1^H} NMR (100.61 MHz, CDCl_3_): *δ* 38.80 and 43.33 (both s, NMe_2_), 62.17 (s, OMe), 120.88, 124.35, 124.87 and 129.42 (four s, C2, C4–C6), 133.54 (s, C3), 147.65 (s, CH=N), 154.29 (s, C1), 187.48 (s, C=S) ppm. IR (thin film, *v*/cm^−1^): 686(w), 696(w), 793(m), 905(w), 923(w), 936(w), 985(w), 1001(w), 1054(s), 1125(s), 1155(s), 1176(s), 1232(s), 1267(m), 1289(s), 1346(w), 1396(s), 1443(m), 1483(m), 1535(br, s) (OC(S)N), 1575(m), 1607(w), 2817(vw), 2899(w), 2938(m), 2990(w), 3065(vw). Anal. Calcd for C_11_H_14_N_2_O_2_S: C, 55.44; H, 5.92; N, 11.76. Found: C, 55.54; H, 5.87; N, 11.67%.

***O*-{3-[(Butylimino)methyl]phenyl} dimethylthiocarbamate 4b**


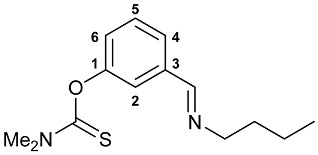


The compound was obtained by **method B** from aldehyde **1** (0.400 g, 1.911 mmol) and *n*-butylamine (0.140 g, 1.914 mmol). The content of the starting aldehyde in the crude product was 5%. ^1^H NMR (300.13 MHz, CDCl_3_): *δ* 0.95 (t, 3H, Me, ^3^*J*_HH_ = 7.4 Hz), 1.33–1.45 and 1.63–1.73 (both m, 2H + 2H, CH_2_), 3.34 and 3.46 (both s, 3H + 3H, NMe_2_), 3.61 (dt, 2H, CH_2_N, ^3^*J*_HH_ = 7.0 Hz, ^4^*J*_HH_ = 1.4 Hz), 7.14 (ddd, 1H, H(C4) or H(C6), ^3^*J*_HH_ = 8.0 Hz, ^4^*J*_HH_ = 2.4 Hz, ^4^*J*_HH_ = 1.1 Hz), 7.41–7.48 (m, 2H, H(C5) + H(C2)), 7.57–7.60 (m, 1H, H(C6) or H(C4)), 8.26 (t, 1H, CH=N, ^4^*J*_HH_ = 1.4 Hz) ppm. ^13^C{^1^H} NMR (100.61 MHz, CDCl_3_): *δ* 13.95 (s, Me), 20.45 and 32.95 (both s, CH_2_), 38.77 and 43.28 (both s, NMe_2_), 61.36 (s, CH_2_N), 121.73, 125.01, 125.83 and 129.22 (four s, C2, C4–C6), 137.78 (s, C3), 154.35 (s, C1), 159.72 (s, CH=N), 187.52 (s, C=S) ppm. IR (thin film, *v*/cm^−1^): 686(w), 697(w), 736(vw), 794(w), 912(vw), 972(w), 1001(w), 1059(w), 1122(m), 1152(s), 1175(s), 1238(s), 1265(m), 1291(s), 1396(s), 1446(m), 1482(m), 1534(br, s) (OC(S)N), 1584(m), 1647(m) (*ν*C=N), 2871(m), 2931(m), 2956(m), 3050(vw).

***O*-{3-[(Benzylimino)methyl]phenyl} dimethylthiocarbamate 4c**


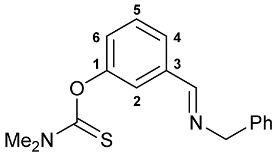


The compound was obtained by **method B** from aldehyde **1** (0.410 g, 1.959 mmol) and benzylamine (0.210 g, 1.960 mmol). The content of the starting aldehyde in the crude product was 6%. ^1^H NMR (400.13 MHz, CDCl_3_): *δ* 3.36 and 3.48 (both s, 3H + 3H, NMe_2_), 4.85 (s, 2H, CH_2_), 7.19 (dd, 1H, H(C4) or H(C6), ^3^*J*_HH_ = 8.2 Hz, ^4^*J*_HH_ = 2.5 Hz), 7.27–7.32 (m, 1H, H_Ar_), 7.35–7.40 (m, 4H, H_Ar_), 7.45–7.49 (m, 1H, H_Ar_), 7.57 (s, 1H, H(C2)), 7.67 (d, 1H, H(C6) or H(C4), ^3^*J*_HH_ = 7.7 Hz), 8.41 (s, 1H, CH=N) ppm. ^13^C{^1^H} NMR (100.61 MHz, CDCl_3_): *δ* 38.81 and 43.34 (both s, NMe_2_), 64.98 (s, CH_2_), 122.03, 125.40, 126.16, 127.11, 128.10, 128.58 and 129.33 (seven s, C2, C4–C6, *o*-C, *m*-C and *p*-C in Ph), 137.61 and 139.11 (both s, C3 and *ipso*-C in Ph), 154.40 (s, C1), 160.97 (s, CH=N), 187.56 (s, C=S) ppm. IR (thin film, *v*/cm^−1^): 698(m), 736(w), 792(w), 913(vw), 1001(w), 1028(w), 1080(w), 1122(s), 1149(s), 1174(s), 1237(s), 1264(m), 1290(s), 1342(w), 1396(s), 1446(m), 1483(m), 1496(m), 1537(br, s) (OC(S)N), 1584(m), 1644(m) (*ν*C=N), 2851(w), 2937(w), 3028(w), 3062(w).

***O*-{3-[(Methoxyimino)methyl]phenyl} diethylthiocarbamate 5a**


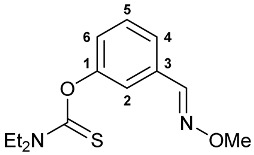


The compound was obtained by **method A** from aldehyde **2** (0.237 g, 0.999 mmol), *O*-methylhydroxylamine hydrochloride (0.100 g, 1.197 mmol), and pyridine (0.30 mL, 3.724 mmol). Yield: 0.246 g (92%). ^1^H NMR (300.13 MHz, CDCl_3_): *δ* 1.32–1.37 (m, 6H, Me in NEt_2_), 3.71 and 3.92 (both q, 2H + 2H, CH_2_ in NEt_2_, ^3^*J*_HH_ = 7.1 Hz), 3.99 (s, 3H, OMe), 7.08–7.12 (m, 1H, H_Ar_), 7.35 (br. s, 1H, H(C2)), 7.38–7.47 (m, 2H, H_Ar_), 8.06 (s, 1H, CH=N) ppm. ^13^C{^1^H} NMR (100.61 MHz, CDCl_3_): *δ* 11.65 and 13.39 (both s, Me in NEt_2_), 44.14 and 48.26 (both s, CH_2_ in NEt_2_), 61.97 (s, OMe), 120.70, 124.18, 124.64 and 129.20 (four s, C2, C4–C6), 133.34 (s, C3), 147.52 (s, CH=N), 154.01 (s, C1), 186.39 (s, C=S) ppm. IR (thin film, *v*/cm^−1^): 693(w), 772(w), 791(w), 885(w), 917(w), 965(w), 979(w), 1001(w), 1054(m), 1079(w), 1095(w), 1120(m), 1150(m), 1160(m), 1168(m), 1222(s), 1247(w), 1286(m), 1316(m), 1348(w), 1361(w), 1380(w), 1429(m), 1443(m), 1458(w), 1487(w), 1513(br, s) (OC(S)N), 1576(w), 1607(w), 2818(vw), 2899(w), 2936(w), 2977(w), 3067(vw). Anal. Calcd for C_13_H_18_N_2_O_2_S: C, 58.62; H, 6.81; N, 10.52. Found: 58.76; H, 7.00; N, 10.48%.

***O*-{3-[(Butylimino)methyl]phenyl} diethylthiocarbamate 5b**


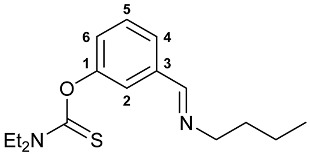


The compound was obtained by **method B** from aldehyde **2** (0.260 g, 1.096 mmol) and *n*-butylamine (0.080 g, 1.094 mmol). The content of the starting aldehyde in the crude product was 1%. ^1^H NMR (300.13 MHz, CDCl_3_): *δ* 0.96 (t, 3H, Me in *^n^*Bu, ^3^*J*_HH_ = 7.4 Hz), 1.32–1.46 (m, 8H, Me in NEt_2_ + CH_2_ in *^n^*Bu), 1.64–1.74 (m, 2H, CH_2_ in *^n^*Bu), 3.60–3.65 (m, 2H, CH_2_N in *^n^*Bu), 3.71 and 3.92 (both q, 2H + 2H, CH_2_ in NEt_2_, ^3^*J*_HH_ = 7.1 Hz), 7.15 (ddd, 1H, H(C4) or H(C6), ^3^*J*_HH_ = 8.0 Hz, ^4^*J*_HH_ = 2.4 Hz, ^4^*J*_HH_ = 1.2 Hz), 7.41–7.49 (m, 2H, H(C5) + H(C2)), 7.57–7.60 (m, 1H, H(C6) or H(C4)), 8.28 (t, 1H, CH=N, ^4^*J*_HH_ = 1.4 Hz) ppm. ^13^C{^1^H} NMR (100.61 MHz, CDCl_3_): *δ* 11.84, 13.56 and 13.92 (three s, Me in NEt_2_ and *^n^*Bu), 20.46 and 32.94 (both s, CH_2_ in *^n^*Bu), 44.31 and 48.41 (both s, CH_2_ in NEt_2_), 61.44 (s, CH_2_N in *^n^*Bu), 121.73, 125.05, 125.85 and 129.21 (four s, C2, C4–C6), 137.75 (s, C3), 154.25 (s, C1), 159.84 (s, CH=N), 186.65 (s, C=S) ppm. IR (thin film, *v*/cm^−1^): 693(w), 771(w), 792(w), 890(w), 930(vw), 964(w), 1001(w), 1023(vw), 1080(w), 1095(w), 1118(m), 1148(m), 1167(s), 1224(s), 1256(m), 1268(m), 1286(s), 1316(m), 1349(w), 1361(m), 1379(m), 1429(s), 1444(s), 1482(m), 1508(br, s) (OC(S)N), 1584(m), 1606(vw), 1647(m) (*ν*C=N), 2872(w), 2932(m), 2958(m), 3073(vw).

***O*-{3-[(Benzylimino)methyl]phenyl} diethylthiocarbamate 5c**


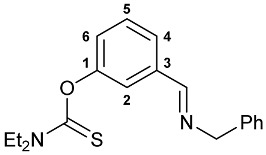


The compound was obtained by **method B** from aldehyde **2** (0.246 g, 1.037 mmol) and benzylamine (0.111 g, 1.036 mmol). The crude product contained a trace amount of the starting aldehyde. Mp: 54–57 °C (dec.). ^1^H NMR (300.13 MHz, CDCl_3_): *δ* 1.25–1.30 (m, 6H, Me in NEt_2_), 3.65 and 3.84 (both q, 2H + 2H, CH_2_ in NEt_2_, ^3^*J*_HH_ = 7.1 Hz), 4.77 (s, 2H, CH_2_), 7.10 (dd, 1H, H(C4) or H(C6), ^3^*J*_HH_ = 8.1 Hz, ^4^*J*_HH_ = 2.4 Hz), 7.19–7.33 (m, 5H, H_Ar_), 7.37–7.42 (m, 1H, H_Ar_), 7.48 (br. s, 1H, H(C2)), 7.58 (d, 1H, H(C6) or H(C4), ^3^*J*_HH_ = 7.6 Hz), 8.34 (s, 1H, CH=N) ppm. ^13^C{^1^H} NMR (100.61 MHz, CDCl_3_): *δ* 11.85 and 13.58 (both s, Me in NEt_2_), 44.33 and 48.44 (both s, CH_2__B_ NEt_2_), 65.00 (s, CH_2_N), 122.01, 125.41, 126.12, 127.09, 128.10, 128.56 and 129.27 (seven s, C2, C4–C6, *o*-, *m*- and *p*-C in Ph), 137.56 and 139.04 (both s, C3 and *ipso*-C in Ph), 154.28 (s, C1), 161.04 (s, CH=N), 186.64 (s, C=S) ppm. IR (KBr, *v*/cm^−1^): 695(m), 734(w), 773(w), 798(w), 890(w), 977(w), 1001(w), 1030(w), 1082(w), 1121(m), 1146(m), 1168(s), 1225(s), 1265(w), 1287(m), 1315(m), 1344(m), 1360(w), 1376(w), 1430(s), 1443(m), 1479(w), 1518(br, s) (OC(S)N), 1581(w), 1601(vw), 1639(m) (*ν*C=N), 2827(w), 2869(w), 2935(w), 2978(m), 3025(w), 3062(w).

***O*-{3-[1-(Methoxyimino)ethyl]phenyl} dimethylthiocarbamate 6**


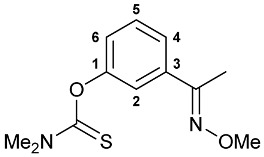


The compound was obtained by **method C** from ketone **3** (0.560 g, 2.508 mmol), *O*-methylhydroxylamine hydrochloride (0.251 g, 3.005 mmol), and pyridine (0.80 mL, 9.930 mmol). Yield: 0.580 g (92%). Mp: 83–84 °C. ^1^H NMR (300.13 MHz, CDCl_3_): *δ* 2.23 (s, 3H, Me), 3.37 and 3.48 (both s, 3H + 3H, NMe_2_), 4.01 (s, 3H, OMe), 7.09 (ddd, 1H, H(C4) or H(C6), ^3^*J*_HH_ = 8.1 Hz, ^4^*J*_HH_ = 2.3 Hz, ^4^*J*_HH_ = 1.1 Hz), 7.38–7.43 (m, 2H, H(C2) + H(C5)), 7.53–7.57 (m, 1H, H(C6) or H(C4)) ppm. ^13^C{^1^H} NMR (100.61 MHz, CDCl_3_): *δ* 12.50 (s, Me), 38.78 and 43.31 (both s, NMe_2_), 62.05 (s, OMe), 120.33, 123.52, 123.53 and 129.04 (four s, C2, C4–C6), 137.86 (s, C3), 153.59 and 154.10 (both s, C=N and C1), 187.64 (s, C=S) ppm. IR (KBr, *v*/cm^−1^): 631(vw), 685(w), 763(w), 794(w), 864(m), 889(vw), 912(w), 962(vw), 1000(w), 1048(s), 1137(m), 1184(m), 1200(s), 1269(w), 1283(m), 1315(w), 1400(m), 1437(m), 1484(w), 1541(br, m) (OC(S)N), 1604(w), 2818(vw), 2900(w), 2940(w), 2957(w), 2997(vw). Anal. Calcd for C_12_H_16_N_2_O_2_S: C, 57.12; H, 6.39; N, 11.10. Found: C, 57.09; H, 6.49; N, 11.07%.

#### 3.2.3. Solution-Based Synthesis and Characteristics of Pd(II) Pincer Complexes **7**–**9**

**Method A.** A stirred solution of PdCl_2_(NCPh)_2_ (46 mg, 0.120 mm) and the corresponding ligand (0.120 mm) in PhCN (2 mL) was heated at ca. 110 °C (oil bath) for 4–5 h. After cooling to room temperature, hexane (10 mL) was added, and the resulting mixture was stirred at room temperature for 30 min. The precipitate obtained was filtered off, washed with hexane (20 mL), dried in air, and purified by column chromatography on silica gel (eluent: CH_2_Cl_2_–EtOH (20:1 in the case of ligands **4b**,**c**, 30:1 for **5c**, 50:1 for **5b**) to give the target complexes as light-yellow crystalline solids.

**Method B.** A mixture of PdCl_2_(NCPh)_2_ (46 mg, 0.120 mm) and the corresponding ligand (0.120 mm) in MeCN (10 mL) was refluxed for 1 h. After cooling to room temperature, the solvent was removed under reduced pressure. The residue obtained was purified by column chromatography on silica gel (eluent: CH_2_Cl_2_–EtOH (20:1 for **4a** or 30:1 for **5a**)) to give the target complexes as light-yellow crystalline solids.

**Method C.** A suspension of PdCl_2_(NCPh)_2_ (46 mg, 0.120 mm) in MeCN (5 mL) or its solution in CH_2_Cl_2_ (4 mL) was slowly added dropwise to a solution of the corresponding ligand (0.120 mm) in MeCN (5 mL) or CH_2_Cl_2_ (4 mL). The reaction mixture was left under ambient conditions until discoloration: 2 h for ligand **6** and 1 day for ligands **4a** and **5a**. The solvent was removed under reduced pressure. In the case of ligand **6**, the residue obtained was rinsed with Et_2_O and dried in air to give the target complex as a light-yellow crystalline solid. In the case of ligands **4a** and **5a**, the resulting residue was purified by column chromatography on silica gel (eluent: CH_2_Cl_2_–EtOH (50:1 for **4a** and 100:1 for **5a**)) to give the target complexes as light-yellow crystalline solids.

**[κ^3^-*S,C,N*-(L)Pd(II)Cl] complex 7a**


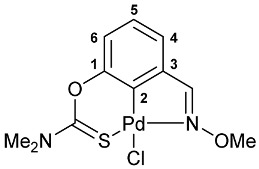


Yield: 74% (**method B**), 91% (**method C**, synthesis in CH_2_Cl_2_). Mp: >215 °C (dec.). ^1^H NMR (300.13 MHz, CDCl_3_): *δ* 3.47 and 3.54 (both s, 3H + 3H, NMe_2_), 4.29 (s, 3H, OMe), 7.00 (dd, 1H, H(C4) or H(C6), ^3^*J*_HH_ = 8.1 Hz, ^4^*J*_HH_ = 1.2 Hz), 7.19–7.24 (m, 1H, H(C5)), 7.28–7.31 (m, 1H, H(C6) or H(C4)), 8.25 (s, 1H, CH=N) ppm. ^13^C{^1^H} NMR (100.61 MHz, CDCl_3_): *δ* 40.50 and 43.56 (both s, NMe_2_), 64.30 (s, OMe), 119.48, 126.16 and 126.42 (three s, C4–C6), 133.70 (s, C3), 141.61 (s, C2), 150.71 (s, C1), 164.31 (s, CH=N), 173.70 (s, C=S) ppm. IR (KBr, *v*/cm^−1^): 643(w), 692(w), 704(w), 788(m), 806(m), 912(w), 951(vw), 967(vw), 984(vw), 1035(s), 1152(w), 1189(w), 1217(m), 1249(s), 1299(s), 1351(m), 1406(m), 1420(m), 1444(m), 1553(br, s) (OC(S)N), 1582(m), 2812(vw), 2926(w), 2998(w), 3047(w). Anal. Calcd for C_11_H_13_ClN_2_O_2_PdS: C, 34.85; H, 3.46; N, 7.39. Found: C, 34.71; H, 3.49; N, 7.33 (**method B**); C, 34.71; H, 3.76; N, 7.49% (**method C**).

**[κ^3^-*S,C,N*-(L)Pd(II)Cl] complex 7b**


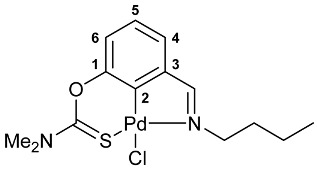


Yield: 83% (**method A**). Mp: >200 °C (dec.). ^1^H NMR (300.13 MHz, CDCl_3_): *δ* 0.96 (t, 3H, Me, ^3^*J*_HH_ = 7.3 Hz), 1.32–1.45 and 1.87–1.96 (both m, 2H + 2H, CH_2_), 3.44 and 3.51 (both s, 3H + 3H, NMe_2_), 3.91 (t, 2H, CH_2_N, ^3^*J*_HH_ = 7.1 Hz), 6.98 (d, 1H, H(C4) or H(C6), ^3^*J*_HH_ = 8.0 Hz), 7.15–7.20 (m, 1 H, H(C5)), 7.31 (d, 1H, H(C6) or H(C4), ^3^*J*_HH_ = 7.1 Hz), 8.06 (s, 1H, CH=N) ppm. ^13^C{^1^H} NMR (100.61 MHz, CDCl_3_): *δ* 13.85 (s, Me), 19.95 and 32.42 (both s, CH_2_), 40.27 and 43.53 (both s, NMe_2_), 59.79 (s, CH_2_N), 119.55, 125.66 and 125.69 (three s, C4–C6), 136.64 (s, C3), 149.38 and 150.96 (both s, C1 and C2), 172.25 (s, CH=N), 174.31 (s, C=S) ppm. IR (KBr, *v*/cm^−1^): 699(w), 782(m), 804(w), 974(vw), 1018(vw), 1063(vw), 1115(vw), 1153(w), 1191(w), 1250(s), 1300(s), 1404(m), 1423(m), 1446(m), 1556(br, s) (OC(S)N), 1587(w), 1614(m) (*ν*C=N), 2869(w), 2931(m), 2949(m), 3047(vw). Anal. Calcd for C_14_H_19_ClN_2_OPdS: C, 41.49; H, 4.73; N, 6.91. Found: C, 41.34; H, 4.79; N, 7.01%.

**[κ^3^-*S,C,N*-(L)Pd(II)Cl] complex 7c**


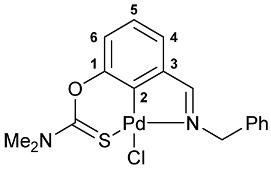


Yield: 71% (**method A**). Mp: >250 °C (dec.). ^1^H NMR (300.13 MHz, (CD_3_)_2_SO): *δ* 3.38 and 3.43 (both s, 3H + 3H, NMe_2_), 5.06 (s, 2H, CH_2_), 6.97 (dd, 1H, H(C4) or H(C6), ^3^*J*_HH_ = 8.2 Hz, ^4^*J*_HH_ = 1.2 Hz), 7.10–7.15 (m, 1H, H_Ar_), 7.20–7.32 (m, 4H, H_Ar_), 7.44–7.47 (m, 2H, *o*-H in Ph), 8.16 (s, 1H, CH=N) ppm. ^13^C{^1^H} NMR (100.61 MHz, (CD_3_)_2_SO): *δ* 40.70 and 43.57 (both s, NMe_2_), 60.44 (s, CH_2_), 120.88, 126.61, 127.42 and 127.98 (four s, C4–C6 and *p*-C in Ph), 128.97 and 129.43 (both s, *o*-C and *m*-C in Ph), 136.78 and 138.30 (both s, C3 and *ipso*-C in Ph), 149.67 and 150.96 (both s, C1 and C2), 172.72 (s, C=S), 176.77 (s, CH=N) ppm. IR (KBr, *v*/cm^−1^): 700(m), 710(w), 761(m), 783(m), 807(w), 917(vw), 1012(w), 1043(w), 1098(vw), 1154(w), 1251(s), 1303(s), 1373(w), 1403(m), 1422(m), 1446(m), 1494(w), 1543(br, s) (OC(S)N), 1584(w), 1610(m) and 1637(w) (both *ν*C=N), 2921(vw), 3045(vw). Anal. Calcd for C_17_H_17_ClN_2_OPdS: C, 46.48; H, 3.90; N, 6.38. Found: C, 46.41; H, 3.98; N, 6.44%.

**[κ^3^-*S,C,N*-(L)Pd(II)Cl] complex 8a**


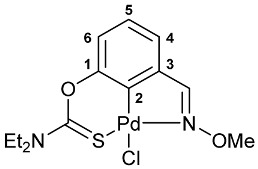


Yield: 75% (**method B**), 76% (**method C**, synthesis in CH_2_Cl_2_). Mp: >210 °C (dec.). ^1^H NMR (300.13 MHz, CDCl_3_): *δ* 1.37 and 1.41 (both t, 3H + 3H, Me in NEt_2_, ^3^*J*_HH_ = 7.2 Hz), 3.76 and 3.96 (both q, 2H + 2H, CH_2_ in NEt_2_, ^3^*J*_HH_ = 7.2 Hz), 4.30 (s, 3H, OMe), 6.99 (dd, 1H, H(C4) or H(C6), ^3^*J*_HH_ = 8.1 Hz, ^4^*J*_HH_ = 1.3 Hz), 7.23 (dd, 1H, H(C5), ^3^*J*_HH_ = 8.1 Hz, ^3^*J*_HH_ = 7.3 Hz), 7.32 (dd, 1H, H(C6) or H(C4), ^3^*J*_HH_ = 7.3 Hz, ^4^*J*_HH_ = 1.3 Hz), 8.28 (s, 1H, CH=N) ppm. ^13^C{^1^H} NMR (100.61 MHz, CDCl_3_): *δ* 11.73 and 13.48 (both s, Me in NEt_2_), 46.22 and 49.25 (both s, CH_2_ in NEt_2_), 64.33 (s, OMe), 119.23, 126.26 and 126.39 (three s, C4–C6), 133.81 (s, C3), 141.76 (s, C2), 150.71 (s, C1), 164.24 (s, CH=N), 172.97 (s, C=S) ppm. IR (KBr, *v*/cm^−1^): 643(vw), 686(w), 699(w), 780(m), 881(w), 972(w), 999(w), 1029(m), 1081(w), 1096(w), 1145(w), 1159(w), 1224(m), 1258(m), 1287(m), 1311(m), 1348(m), 1386(w), 1438(m), 1460(m), 1525(br, s) (OC(S)N), 1586(vw), 1633(vw), 2931(w), 2974(w), 3051(vw). Anal. Calcd for C_13_H_17_ClN_2_O_2_PdS: C, 38.34; H, 4.21; N, 6.88. Found: C, 38.31; H, 4.28; N, 6.79 (**method B**); C, 38.11; H, 4.21; N, 6.88% (**method C**).

**[κ^3^-*S,C,N*-(L)Pd(II)Cl] complex 8b**


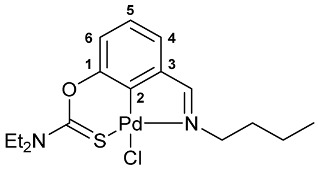


Yield: 85% (**method A**). ^1^H NMR (300.13 MHz, CDCl_3_): *δ* 0.96 (t, 3H, Me in *^n^*Bu, ^3^*J*_HH_ = 7.3 Hz), 1.32–1.44 (m, 8H, Me in NEt_2_ + CH_2_ in *^n^*Bu), 1.87–1.97 (m, 2H, CH_2_ in *^n^*Bu), 3.74 (q, 2H, CH_2_ in NEt_2_, ^3^*J*_HH_ = 7.1 Hz), 3.90–3.97 (m, 4H, CH_2_N in *^n^*Bu + CH_2_ in NEt_2_), 6.98 (d, 1H, H(C4) or H(C6), ^3^*J*_HH_ = 8.2 Hz), 7.18–7.23 (m, 1H, H(C5)), 7.33 (d, 1H, H(C6) or H(C4), ^3^*J*_HH_ = 7.2 Hz), 8.08 (s, 1 H, CH=N) ppm. ^13^C{^1^H} NMR (100.61 MHz, CDCl_3_): *δ* 11.69, 13.49 and 13.84 (three s, Me in NEt_2_ and *^n^*Bu), 19.93 and 32.41 (both s, CH_2_ in *^n^*Bu), 46.19 and 49.18 (both s, CH_2_ in NEt_2_), 59.76 (s, CH_2_N in *^n^*Bu), 119.44, 125.64 and 125.74 (three s, C4–C6), 136.55 (s, C3), 149.38 and 150.88 (both s, C1 and C2), 172.26 (s, CH=N), 173.26 (s, C=S) ppm. IR (KBr, *v*/cm^−1^): 683(w), 701(w), 736(vw), 777(w), 882(w), 973(w), 994(w), 1021(w), 1035(w), 1076(w), 1094(w), 1114(w), 1145(m), 1182(w), 1222(m), 1234(m), 1257(m), 1290(m), 1310(m), 1359(m), 1379(w), 1438(s), 1459(m), 1526(br, s) (OC(S)N), 1587(w), 1614(m) (*ν*C=N), 2859(w), 2869(w), 2931(w), 2955(w), 3051(vw). Anal. Calcd for C_16_H_23_ClN_2_OPdS: C, 44.35; H, 5.35; N, 6.47. Found: C, 43.98; H, 5.50; N, 6.28%.

**[κ^3^-*S,C,N*-(L)Pd(II)Cl] complex 8c**


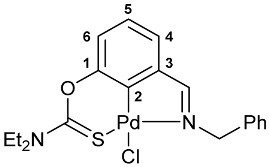


Yield: 80% (**method A**). ^1^H NMR (300.13 MHz, CDCl_3_): *δ* 1.33–1.43 (m, 6H, Me in NEt_2_), 3.74 and 3.94 (both q, 2H + 2H, CH_2_ in NEt_2_, ^3^*J*_HH_ = 7.1 Hz), 5.24 (s, 2H, CH_2_), 6.98 (d, 1H, H(C4) or H(C6), ^3^*J*_HH_ = 8.0 Hz), 7.14–7.19 (m, 1H, H(C5)), 7.24–7.31 (m, 2H, H_Ar_), 7.33–7.42 (m, 3H, H_Ar_), 7.49 (d, 1H, H(C6) or H(C4), ^3^*J*_HH_ = 7.2 Hz), 7.94 (s, 1H, CH=N) ppm. ^13^C{^1^H} NMR (100.61 MHz, CDCl_3_): *δ* 11.53 and 13.33 (both s, Me in NEt_2_), 46.05 and 49.05 (both s, CH_2_ in NEt_2_), 61.51 (s, CH_2_), 119.49, 125.55, 125.90 and 127.84 (four s, C4–C6 and *p*-C in Ph), 128.74 and 129.60 (both s, *o*-C and *m*-C in Ph), 136.08 and 136.44 (both s, C3 and *ipso*-C in Ph), 149.20 and 150.60 (both s, C2 and C1), 172.96 (s, CH=N), 173.01 (s, C=S) ppm. IR (KBr, *v*/cm^−1^): 703(w), 760(w), 777(w), 796(w), 881(vw), 996(w), 1041(vw), 1082(w), 1146(w), 1182(vw), 1224(m), 1233(m), 1261(m), 1290(m), 1308(m), 1358(w), 1373(w), 1439(m), 1493(w), 1529(br, s) (OC(S)N), 1585(vw), 1608(w) (*ν*C=N), 2870(vw), 2934(vw), 2973(w), 3057(vw). Anal. Calcd for C_19_H_21_ClN_2_OPdS: C, 48.83; H, 4.53; N, 5.99. Found: C, 48.76; H, 4.60; N, 6.02%.

**[κ^3^-*S,C,N*-(L)Pd(II)Cl] complex 9**


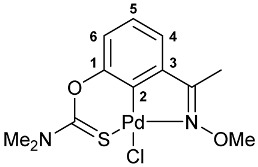


Yield: 88% (**method C**, synthesis in MeCN). Mp: >230 °C (dec.). ^1^H NMR (300.13 MHz, CDCl_3_): *δ* 2.48 (s, 3H, Me), 3.49 and 3.55 (both s, 3H + 3H, NMe_2_), 4.22 (s, 3H, OMe), 7.02 (dd, 1H, H(C4) or H(C6), ^3^*J*_HH_ = 8.1 Hz, ^4^*J*_HH_ = 1.2 Hz), 7.19–7.24 (m, 1H, H(C5)), 7.32 (dd, 1H, H(C6) or H(C4), ^3^*J*_HH_ = 7.5 Hz, ^4^*J*_HH_ = 1.2 Hz) ppm. ^13^C{^1^H} NMR (100.61 MHz, CDCl_3_): *δ* 12.59 (s, Me), 40.38 and 43.52 (both s, NMe_2_), 63.33 (s, OMe), 119.62, 125.29 and 125.66 (three s, C4–C6), 134.88 (s, C3), 144.32 (s, C2), 150.70 (s, C1), 173.93 and 175.17 (both s, C=N and C=S) ppm. IR (KBr, *v*/cm^−1^): 620(vw), 662(vw), 701(w), 782(m), 873(m), 935(vw), 966(w), 1005(w), 1041(m), 1130(w), 1158(w), 1255(m), 1301(s), 1334(m), 1372(w), 1408(m), 1422(m), 1551(br, s) (OC(S)N), 1588(w), 1624(vw), 2816(vw), 2929(w), 2999(vw), 3058(vw). Anal. Calcd for C_12_H_15_ClN_2_O_2_PdS: C, 36.66; H, 3.85; N, 7.12. Found: C, 36.51; H, 4.01; N, 7.28%.

#### 3.2.4. Solid-Phase Synthesis of Complexes **7a**, **8a**, and **9**

PdCl_2_(NCPh)_2_ (46 mg, 0.120 mmol) and the corresponding ligand (0.120 mmol) were manually ground in an agate mortar for 10–15 min, alternating periods of grinding with breaks and scraping the reaction mixture with a spatula.

**Method A.** In the case of ligand **6**, the resulting brown oily residue was kept in a closed glass tube with a paper indicator, analyzing the reaction course by IR and NMR spectroscopy. The paper indicator showed intensive release of HCl during the first several days, confirming the occurrence of metalation. The IR spectroscopic monitoring revealed almost complete cyclopalladation in 2 weeks (see Figure 3 and Figures S15, S18, S27–S31 in the Supporting Information). During this period of time, the oily residue turned into a beige free-flowing powder. Anal. Calcd for C_12_H_15_ClN_2_O_2_PdS·0.25PhCN: C, 39.42; H, 3.91; N, 7.52. Found: C, 39.58; H, 4.28; N, 7.50%. The ^1^H NMR spectrum of the resulting sample in CDCl_3_, recorded directly after dissolution, confirmed the formation of over 95% of pincer complex **9** and the presence of residual PhCN (Figure S32 in the Supporting Information).

**Method B.** In the case of ligands **4a** and **5a**, the resulting brown residue was heated in an open test tube at 105–107 °C (for **4a**) or 85–90 °C (for **5a**) for 15 min to give a non-uniform dark-yellow free-flowing powder. The optimal reaction temperature corresponded to intensive release of HCl detected with a paper indicator during preliminary heating the ground sample in a melting point measuring apparatus. The IR spectroscopic studies confirmed the occurrence of cyclopalladation upon heating (see, for example, Figures S23–S26 in the Supporting Information). The sample obtained after heating was dissolved in CH_2_Cl_2_ and purified immediately by column chromatography on silica gel (eluent: CH_2_Cl_2_–EtOH (50:1 for **4a**, 100:1 for **5a**)) to give the target pincer complexes as light-yellow crystalline solids. **Complex 7a.** Yield: 94%. Anal. Calcd for C_11_H_13_ClN_2_O_2_PdS: C, 34.85; H, 3.46; N, 7.39. Found: C, 35.20; H, 3.52; N, 7.33%. **Complex 8a.** Yield: 89%. Anal. Calcd for C_13_H_17_ClN_2_O_2_PdS: C, 38.24; H, 4.21; N, 6.88. Found: C, 38.31; H, 4.33; N, 6.88%.

**Method C.** Alternatively, the brown residue obtained by grinding ligand **4a** or **5a** with the Pd(II) precursor in a mortar (which does not contain the pincer product according to the results of IR spectroscopic studies (Figure S25 in the Supporting Information) was dissolved in CH_2_Cl_2_ and purified immediately by column chromatography on silica gel (eluent: CH_2_Cl_2_–EtOH (100:1)) to give the target pincer complexes as light-yellow crystalline solids. **Complex 7a.** Yield: 67%. Anal. Calcd for C_11_H_13_ClN_2_O_2_PdS: C, 34.85; H, 3.46; N, 7.39. Found: C, 35.28; H, 3.57; N, 7.47%. **Complex 8a.** Yield: 65%. Anal. Calcd for C_13_H_17_ClN_2_O_2_PdS: C, 38.34; H, 4.21; N, 6.88. Found: C, 38.29; H, 4.30; N, 6.88%.

### 3.3. X-Ray Crystallography

X-ray diffraction data for compounds **7a**, **7c**, and **9** were collected at 220 K with a Bruker APEXII Quazar CCD diffractometer (Bruker AXS GmbH, Karlsruhe, Germany), using graphite monochromated Mo-Kα radiation (λ = 0.71073 Å). Using Olex2 v. 1.5 [31], the structures were solved with the ShelXT 2018/2 [32] structure solution program using Intrinsic Phasing and refined with the XL 2008 [33] refinement package using Least Squares minimization against F^2^_hkl_ in anisotropic approximation for non-hydrogen atoms. Positions of hydrogen atoms were calculated, and they were refined in the isotropic approximation within the riding model. Crystal data and structure refinement parameters are given in Table 3. CCDC 2,517,175 (**7a**), 2,517,173 (**7c**) and 2,517,174 (**9**) contain the supplementary crystallographic data for this paper.

### 3.4. Cytotoxicity Studies

The cytotoxic activity of the compounds obtained was investigated on human colorectal carcinoma (HCT116), breast cancer (MCF7), prostate adenocarcinoma (PC3), chronic myelogenous leukemia (K562 and K562/iS9), multiple plasmacytoma (AMO1), and acute lymphoblastic leukemia (H9) cell lines, as well as human embryonic kidney (HEK293) and mammary epithelial (HBL100 and HBL100/Dox) cells used as non-cancerous cell lineages. All the cell lines were obtained from American Type Culture Collection (ATCC) (Manassas, VA, USA). The tested compounds were initially dissolved in DMSO. Cisplatin was obtained from a commercial source (as an infusion concentrate in natural saline solution). The experiments were performed using the conventional MTT assay (ICN Biomedicals, Eschwege, Germany) according to the previously published procedure [34].

## 4. Conclusions

Thus, a range of new hybrid pincer-type ligands, combining the imine and thiocarbamate ancillary donor moieties, were obtained from readily available *meta*-thiocarbamoyl-substituted benzaldehyde and acetophenone precursors. These compounds were shown to undergo direct cyclopalladation both in solution and under solvent-free conditions, where the most active derivatives, namely, the oxime-based ligands with an OMe substituent at the imine nitrogen atom did not require additional energy supply and smoothly formed the desired Pd(II) pincer complexes upon mechanochemical activation. The resulting palladacycles displayed prominent cytotoxic effects against several solid and hematopoietic cancer cell cultures, stipulated by the coordination with Pd(II) ions, and similar levels of activity against parent and doxorubicin-resistant cell lines, which renders further search for potential anticancer agents among this type of organometallic compounds very promising.

## Figures and Tables

**Table 1:** Cytotoxicities of the complexes obtained against selected human solid cancer and non-cancerous cell lines.

Entry	Compound	IC_50_ ± SD, *^a^* μM					
		HCT116	MCF7	PC3	HEK293	HBL100	HBL100/Dox
1	**7a**	11.5 ± 1.4	24.5 ± 1.0	10.8 ± 0.6	13.0 ± 2.4	17.1 ± 1.0	24.7 ± 0.9
2	**7b**	6.6 ± 0.7	12.8 ± 0.8	3.6 ± 0.1	8.3 ± 0.6	7.7 ± 0.2	14.5 ± 0.8
3	**7c**	4.1 ± 0.5	8.4 ± 1.0	2.2 ± 0.4	5.2 ± 0.7	5.2 ± 1.2	10.9 ± 0.9
4	**8a**	12.0 ± 2.2	16.0 ± 2.0	8.2 ± 1.0	15.0 ± 2.0	19.0 ± 3.0	25.0 ± 1.0
5	**8b**	6.8 ± 0.2	11.1 ± 2.8	4.1 ± 0.1	6.7 ± 0.3	8.8 ± 1.2	14.8 ± 0.6
6	**8c**	4.4 ± 0.6	7.8 ± 0.6	2.5 ± 0.2	6.3 ± 1.1	5.6 ± 0.8	11.7 ± 1.8
7	**9**	13.9 ± 1.5	24.4 ± 1.4	9.9 ± 0.3	14.6 ± 0.4	15.6 ± 0.4	21.9 ± 1.2
8	cisplatin	18.0 ± 2.0	25.0 ± 4.0	16.0 ± 3.0	12.5 ± 1.5	14.6 ± 3.6	23.6 ± 3.6

*^a^* SD is the standard deviation of the value.

**Table 2:** Cytotoxicities of the complexes obtained against selected human hematopoietic cancer cell lines.

Entry	Compound	IC_50_ ± SD, *^a^* μM			
		K562	K562/iS9	AMO1	H9
1	**7a**	5.0 ± 0.6	6.9 ± 0.5	6.1 ± 0.6	5.7 ± 0.2
2	**7b**	1.9 ± 0.3	2.3 ± 0.1	2.6 ± 0.3	1.4 ± 0.2
3	**7c**	1.2 ± 0.2	1.6 ± 0.1	1.3 ± 0.1	1.7 ± 0.1
4	**8a**	3.6 ± 0.4	3.5 ± 0.7	3.2 ± 0.2	2.8 ± 0.2
5	**8b**	2.1 ± 0.8	2.5 ± 0.6	2.1 ± 0.9	1.5 ± 0.3
6	**8c**	1.6 ± 0.4	3.1 ± 0.2	1.2 ± 0.3	2.3 ± 0.2
7	**9**	5.5 ± 0.7	6.4 ± 1.1	4.6 ± 0.4	7.3 ± 0.3
8	cisplatin	15.5 ± 0.5	16.0 ± 2.0	3.2 ± 0.6	3.0 ± 1.0

*^a^* SD is the standard deviation of the value.

**Table 3:** Crystal data and structure refinement parameters for **7a**, **7c** and **9**.

	7a	7c	9
Empirical formula	C_11_H_13_ClN_2_O_2_PdS	C_17_H_17_ClN_2_O_2_PdS	C_12_H_15_ClN_2_O_2_PdS
Formula weight	379.14	439.23	393.17
*T*, K	220	220	220
Crystal system	Monoclinic	Monoclinic	Monoclinic
Space group	*C2*/*c*	*P2_1_*/*c*	*P2_1_*/*c*
Z	8	4	4
a, Å	17.335(9)	12.894(4)	10.696(2)
b, Å	7.626(4)	9.391(3)	7.4476(15)
c, Å	21.696(16)	14.691(4)	18.401(4)
α, °	90	90	90
β, °	109.472(6)	108.432(3)	104.332(2)
γ, °	90	90	90
V, Å^3^	2704(3)	1687.6(8)	1420.1(5)
D_calc_ (g cm^−1^)	1.863	1.729	1.839
Linear absorption, μ (cm^−1^)	17.18	13.86	16.4
F(000)	1504	880	784
2θmax, °	56	50	56
Reflections measured	11,789	2951	12,305
Independent reflections	3254	2951	3423
Observed reflections [I > 2σ(I)]	2374	2517	2451
Parameters	166	211	177
R1	0.0416	0.0963	0.0454
wR2	0.0888	0.2665	0.1239
GOF	1.005	1.145	0.969
Δρ_max_/Δρ_min_ (e Å^−3^)	0.533/−0.808	2.926/−1.258	0.902/−1.571
CCDC	2,517,175	2,517,173	2,517,174

## Data Availability

The data presented in this study are available in the article and Supplementary Materials.

## Supplementary Materials

The following supporting information can be downloaded at https://www.mdpi.com/article/10.3390/molecules31030546/s1: Figures S1–S32: the NMR and IR spectra of the compounds obtained and residues of the solid-phase reactions.
