# Supplementary material for: Imine–Thiocarbamate Hybrid Pincer Systems: From Mechanochemical Activation to Cytotoxicity Evaluation of the Cyclopalladated Derivatives
Source: Molecules. 2026 Feb 4;31(3):546. doi: 10.3390/molecules31030546 (PMC12899013; doi:10.3390/molecules31030546)
Supplement: Supplementary file 1 [file molecules-31-00546-s001.zip › molecules-4103900-supplementary.pdf]

Supporting information

Imine–thiocarbamate hybrid pincer systems: from mechanochemical activation to cytotoxicity evaluation of the cyclopalladated derivatives

Aleksandr A. Spiridonov [1], Diana V. Aleksanyan [1,]* Dmitry V. Yakshin [1], Yulia V. Nelyubina [2], Ekaterina Yu. Rybalkina [3], Zinaida S. Klemenkova [1] and Vladimir A. Kozlov [1]

[1] A. N. Nesmeyanov Institute of Organoelement Compounds, Russian Academy of Sciences, ul. Vavilova 28, str. 1, Moscow, 119334 Russia

[2] Federal Research Center of Problems of Chemical Physics and Medicinal Chemistry, Russian Academy of Sciences, pr. Akademika Semenova 1, Chernogolovka, Moscow Oblast, 142432 Russia

[3] N. N. Blokhin National Medical Research Center of Oncology of the Ministry of Health of the Russian Federation, Kashirskoe shosse 23, Moscow, 115478 Russia

*corresponding author: aleksanyan.diana@ineos.ac.ru

Table of contents

	Page
**Figure S1.** ^{1}H NMR spectrum of ligand **4b** (300.13 MHz, $CDCl_3$)	S3
**Figure S2.** $^{13}C\{^{1}H\}$ NMR spectrum of ligand **4b** (100.61 MHz, $CDCl_3$)	S4
**Figure S3.** IR spectrum of ligand **4b**	S5
**Figure S4.** ^{1}H NMR spectrum of complex **7b** (300.13 MHz, $CDCl_3$)	S6
**Figure S5.** $^{13}C\{^{1}H\}$ NMR spectrum of complex **7b** (100.61 MHz, $CDCl_3$)	S7
**Figure S6.** IR spectrum of complex **7b**	S8
**Figure S7.** ^{1}H NMR spectrum of ligand **5c** (300.13 MHz, $CDCl_3$)	S9
**Figure S8.** $^{13}C\{^{1}H\}$ NMR spectrum of ligand **5c** (100.61 MHz, $CDCl_3$)	S10
**Figure S9.** IR spectrum of ligand **5c**	S11
**Figure S10.** ^{1}H NMR spectrum of complex **8c** (300.13 MHz, $CDCl_3$)	S12
**Figure S11.** $^{13}C\{^{1}H\}$ NMR spectrum of complex **8c** (100.61 MHz, $CDCl_3$)	S13
**Figure S12.** IR spectrum of complex **8c**	S14
**Figure S13.** ^{1}H NMR spectrum of ligand **6** (400.13 MHz, $CDCl_3$)	S15
**Figure S14.** $^{13}C\{^{1}H\}$ NMR spectrum of ligand **6** (100.61 MHz, $CDCl_3$)	S16
**Figure S15.** IR spectrum of ligand **6**	S17
**Figure S16.** ^{1}H NMR spectrum of complex **9** (300.13 MHz, $CDCl_3$)	S18
**Figure S17.** $^{13}C\{^{1}H\}$ NMR spectrum of complex **9** (100.61 MHz, $CDCl_3$)	S19
**Figure S18.** IR spectrum of complex **9**	S20
**Figure S19.** IR spectrum of ligand **4c**	S21
**Figure S20.** IR spectrum of complex **7c**	S22

Figure S21. IR spectrum of the ground mixture of ligand **4c** and $PdCl_2(NCPh)_2$ (in 1 h after grinding) S23
Figure S22. IR spectrum of the solid residue obtained after heating the ground mixture of ligand **4c** and $PdCl_2(NCPh)_2$ (130–135 °C, 10 min) S24
Figure S23. IR spectrum of ligand **4a** S25
Figure S24. IR spectrum of complex **7a** S26
Figure S25. IR spectrum of the ground mixture of ligand **4a** and $PdCl_2(NCPh)_2$ (in 1.5 h after grinding) S27
Figure S26. IR spectrum of the solid residue obtained after heating the ground mixture of ligand **4a** and $PdCl_2(NCPh)_2$ (105–107 °C, 15 min) S28
Figure S27. IR spectrum of the ground mixture of ligand **6** and $PdCl_2(NCPh)_2$ (in 1 h after grinding) S29
Figure S28. IR spectrum of the ground mixture of ligand **6** and $PdCl_2(NCPh)_2$ (in 1 day after grinding) S30
Figure S29. IR spectrum of the ground mixture of ligand **6** and $PdCl_2(NCPh)_2$ (in 2 days after grinding) S31
Figure S30. IR spectrum of the ground mixture of ligand **6** and $PdCl_2(NCPh)_2$ (in 1 week after grinding) S32
Figure S31. IR spectrum of the ground mixture of ligand **6** and $PdCl_2(NCPh)_2$ (in 2 weeks after grinding) S33
Figure S32. ^{1}H NMR spectrum of the free-flowing powder obtained in 2 weeks after grinding ligand **6** and $PdCl_2(NCPh)_2$, registered directly after dissolution in $CDCl_3$ (300 mHz) S34

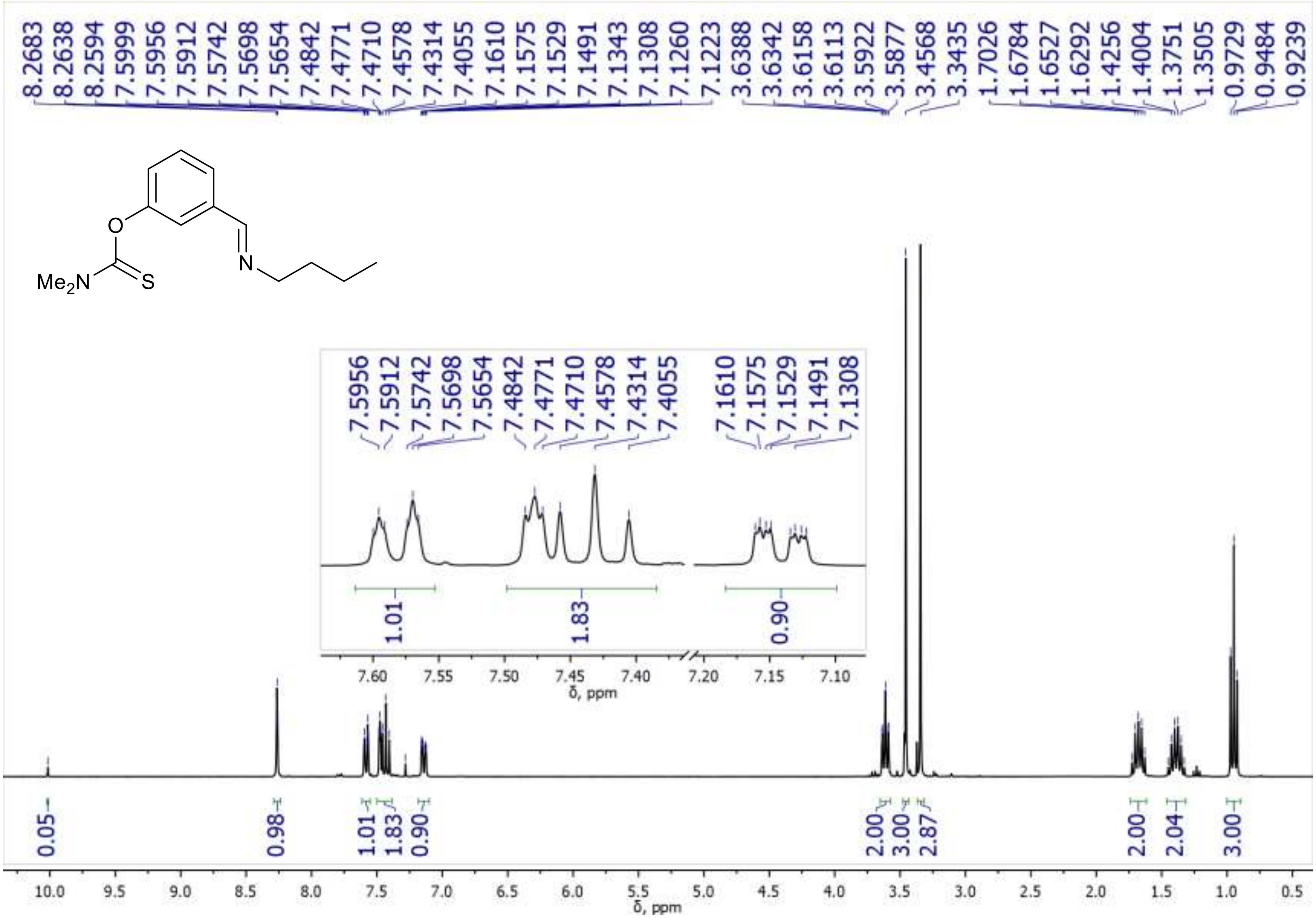

Figure S1. ^{1}H NMR spectrum of ligand **4b** (300.13 MHz, $CDCl_3$)

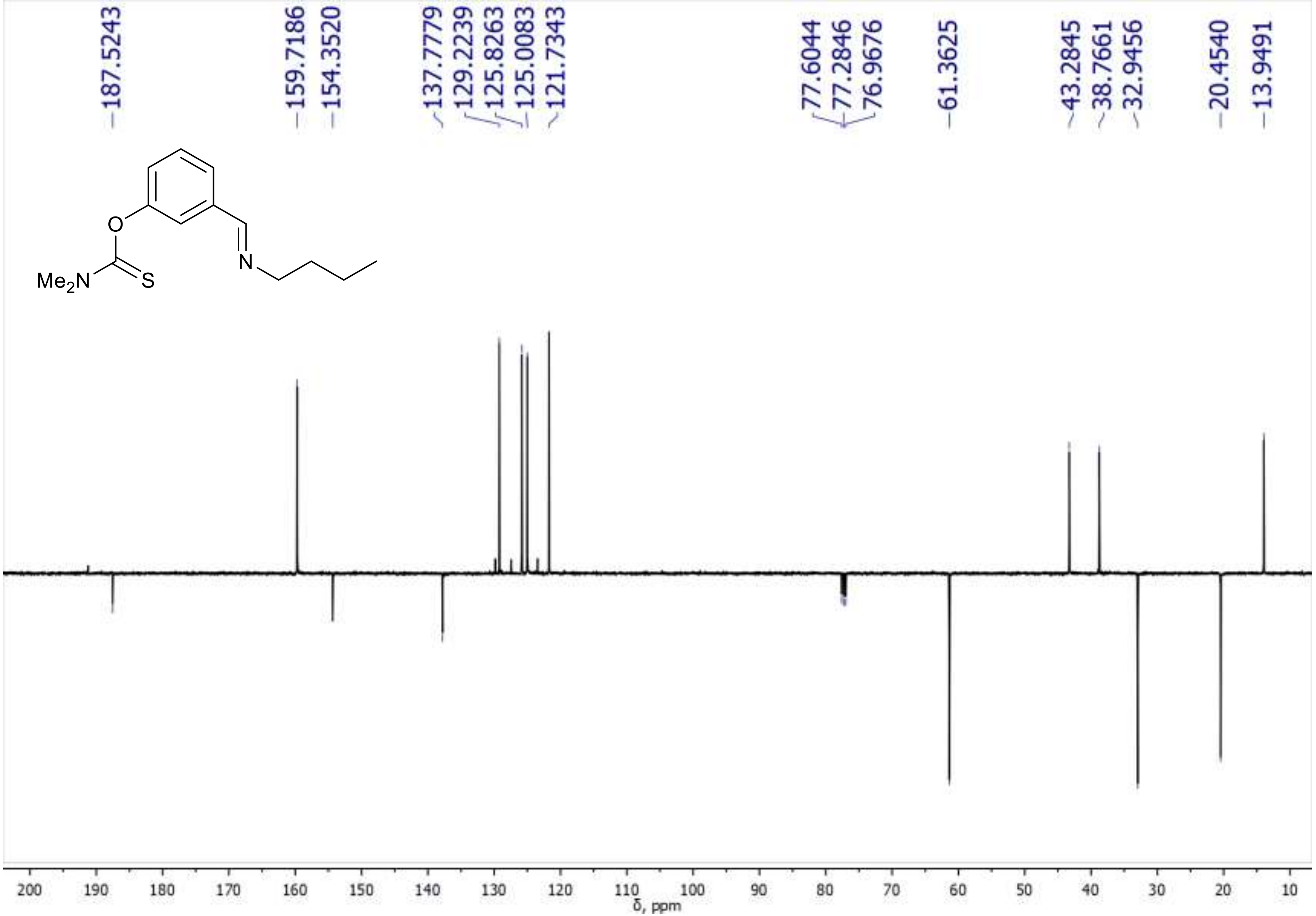

Figure S2. $^{13}C\{^{1}H\}$ NMR spectrum of ligand **4b** (100.61 MHz, $CDCl_3$)

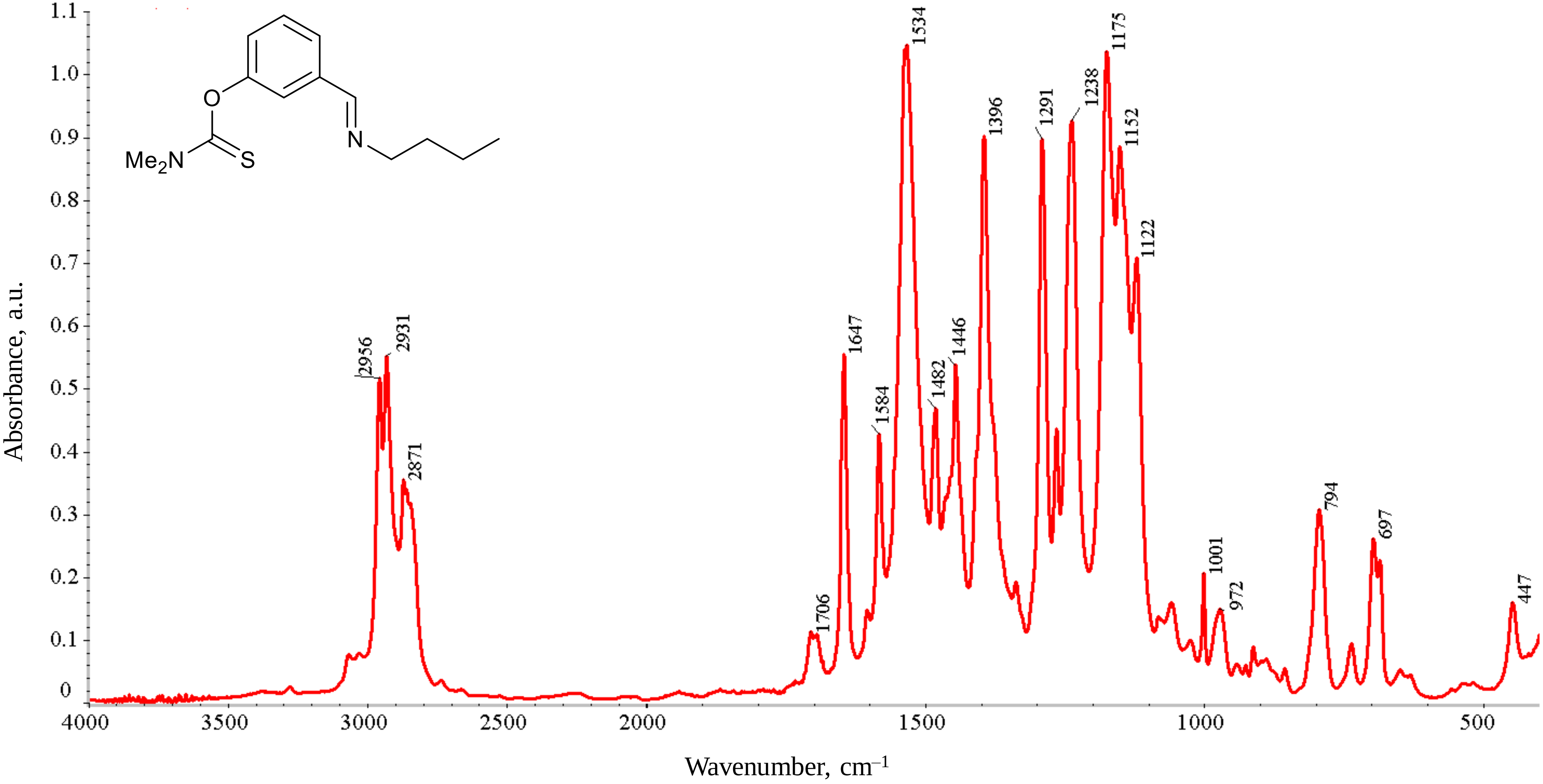

Figure S3. IR spectrum of ligand **4b**

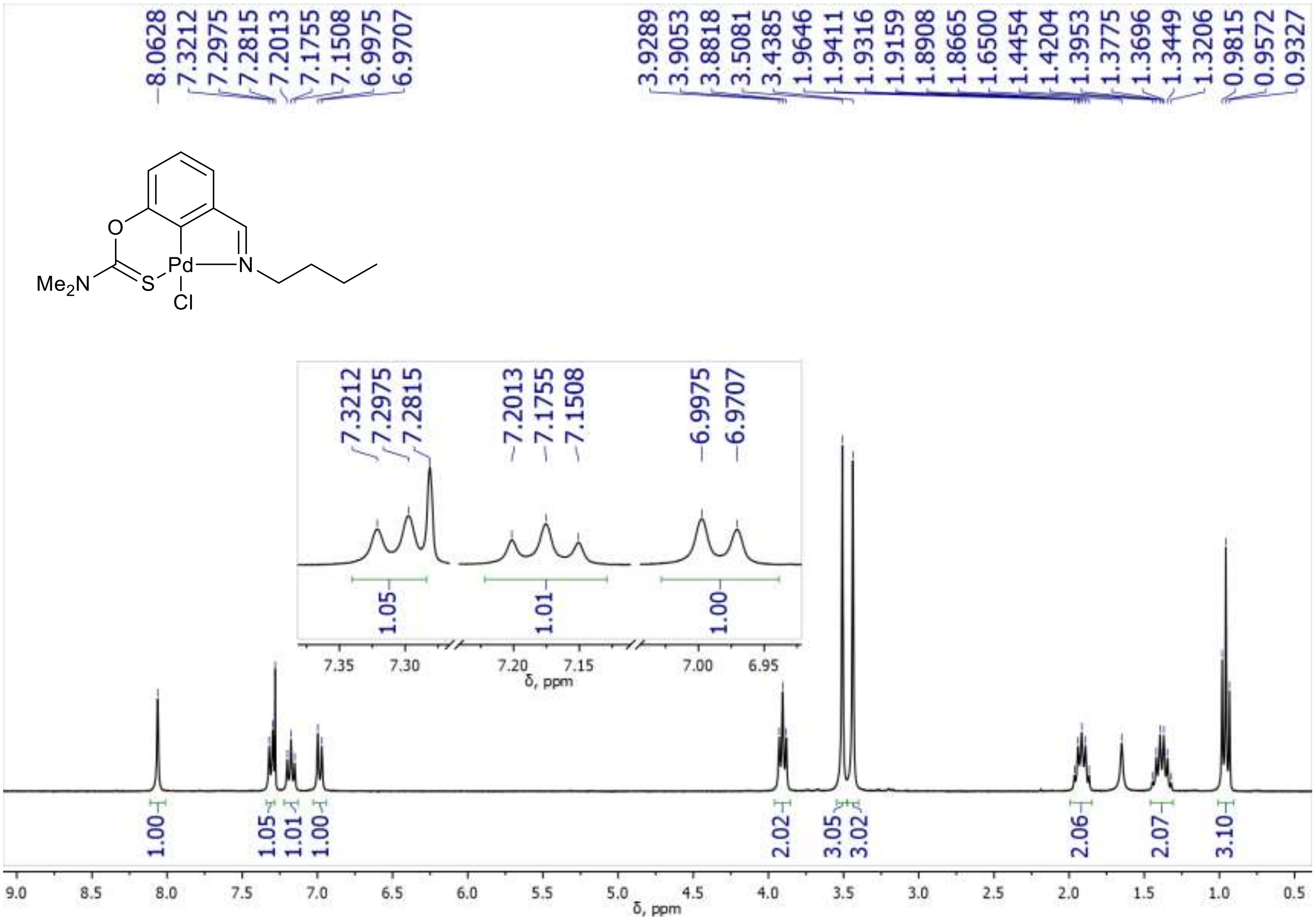

Figure S4. ^{1}H NMR spectrum of complex **7b** (300.13 MHz, $CDCl_3$)

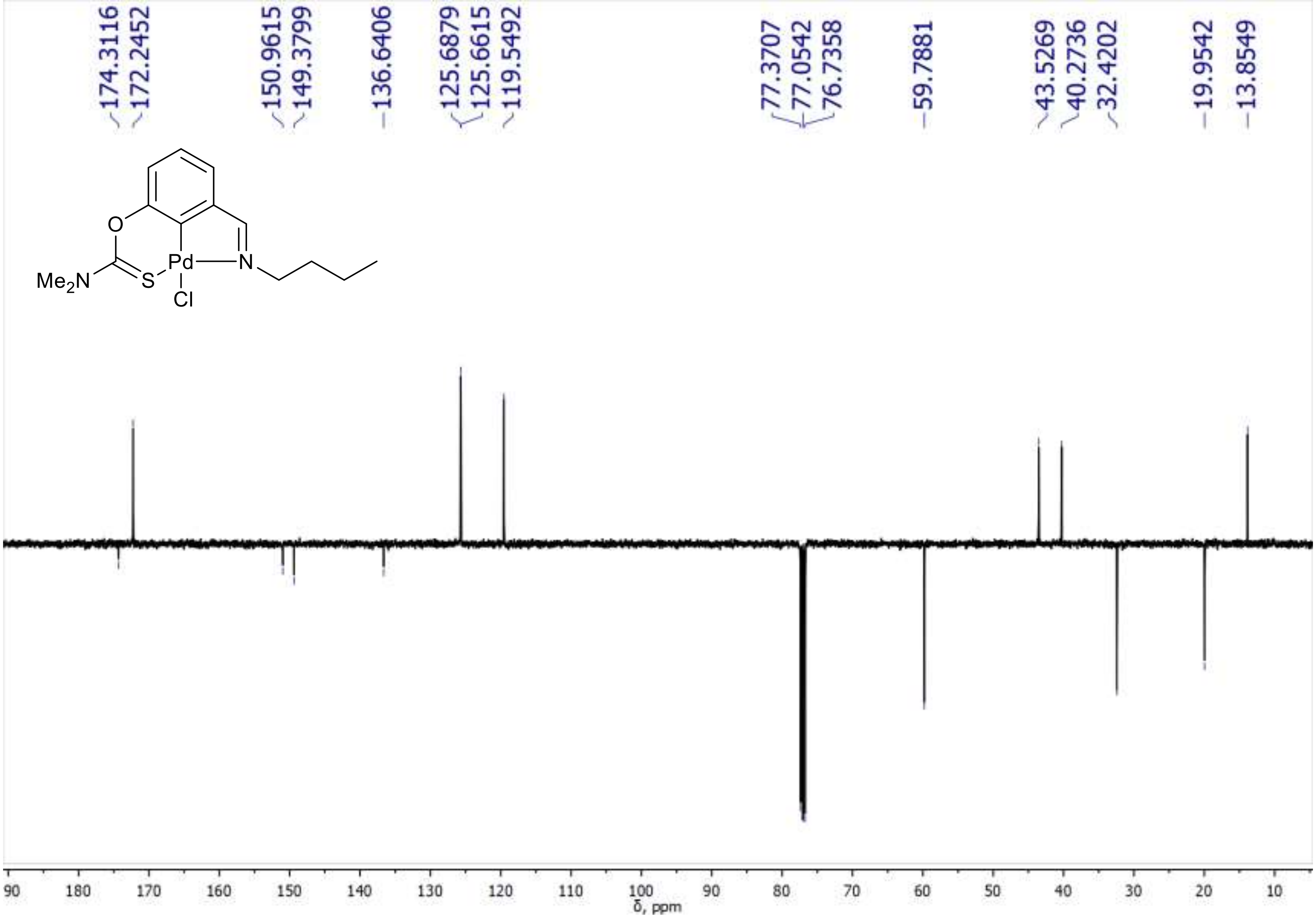

Figure S5. $^{13}C\{^1H\}$ NMR spectrum of complex **7b** (100.61 MHz, $CDCl_3$)

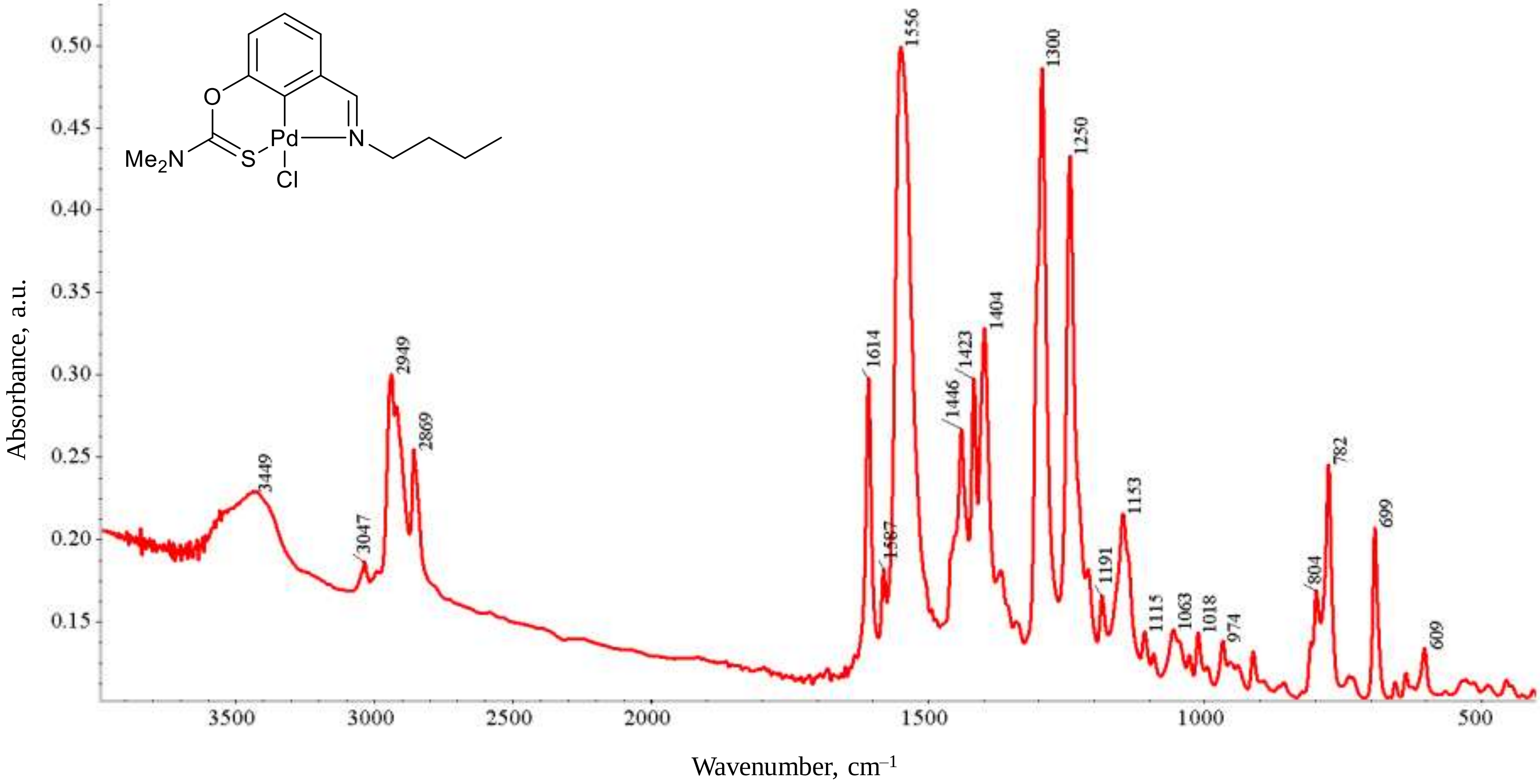

Figure S6. IR spectrum of complex **7b**

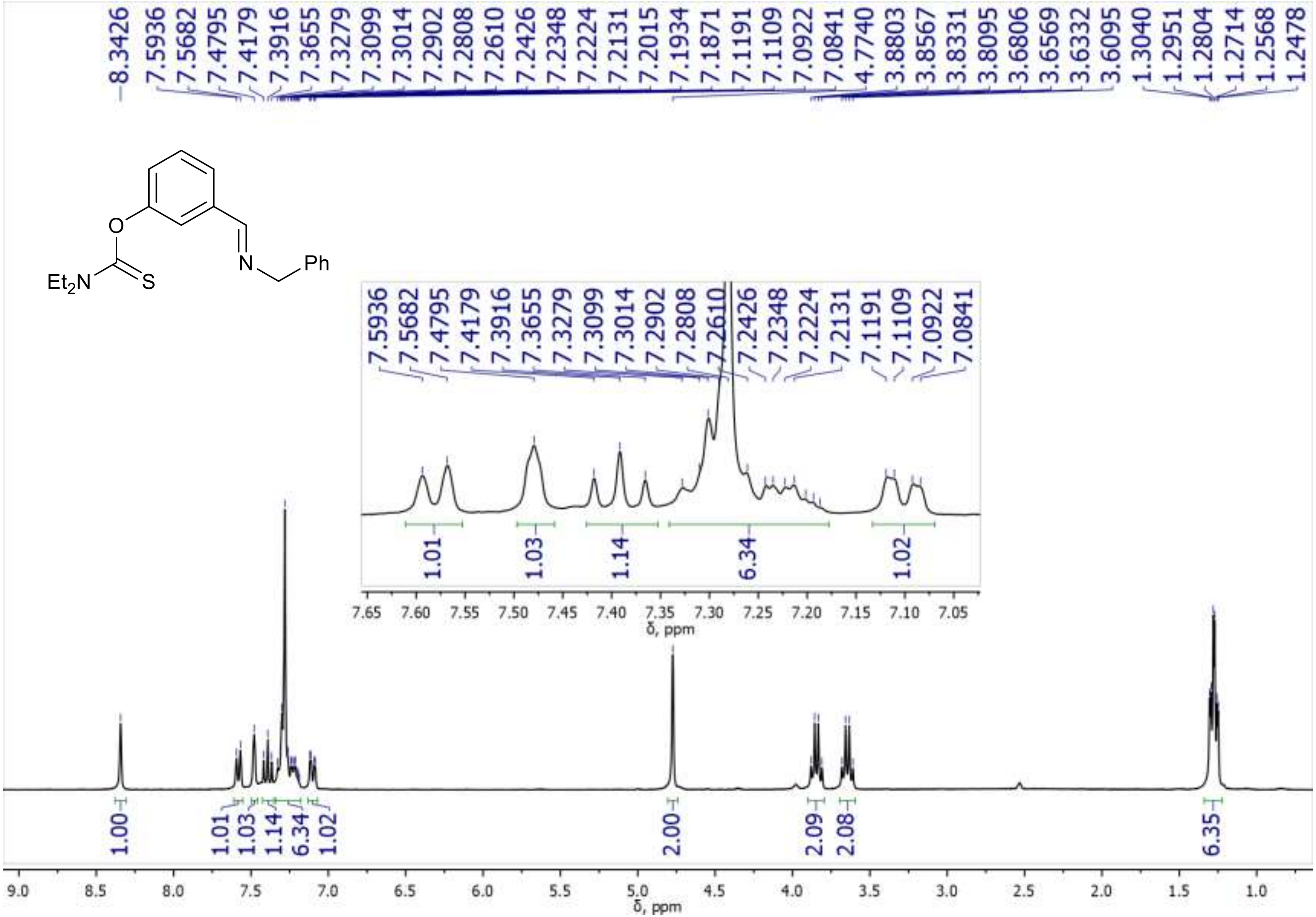

Figure S7. ^{1}H NMR spectrum of ligand **5c** (300.13 MHz, $CDCl_3$)

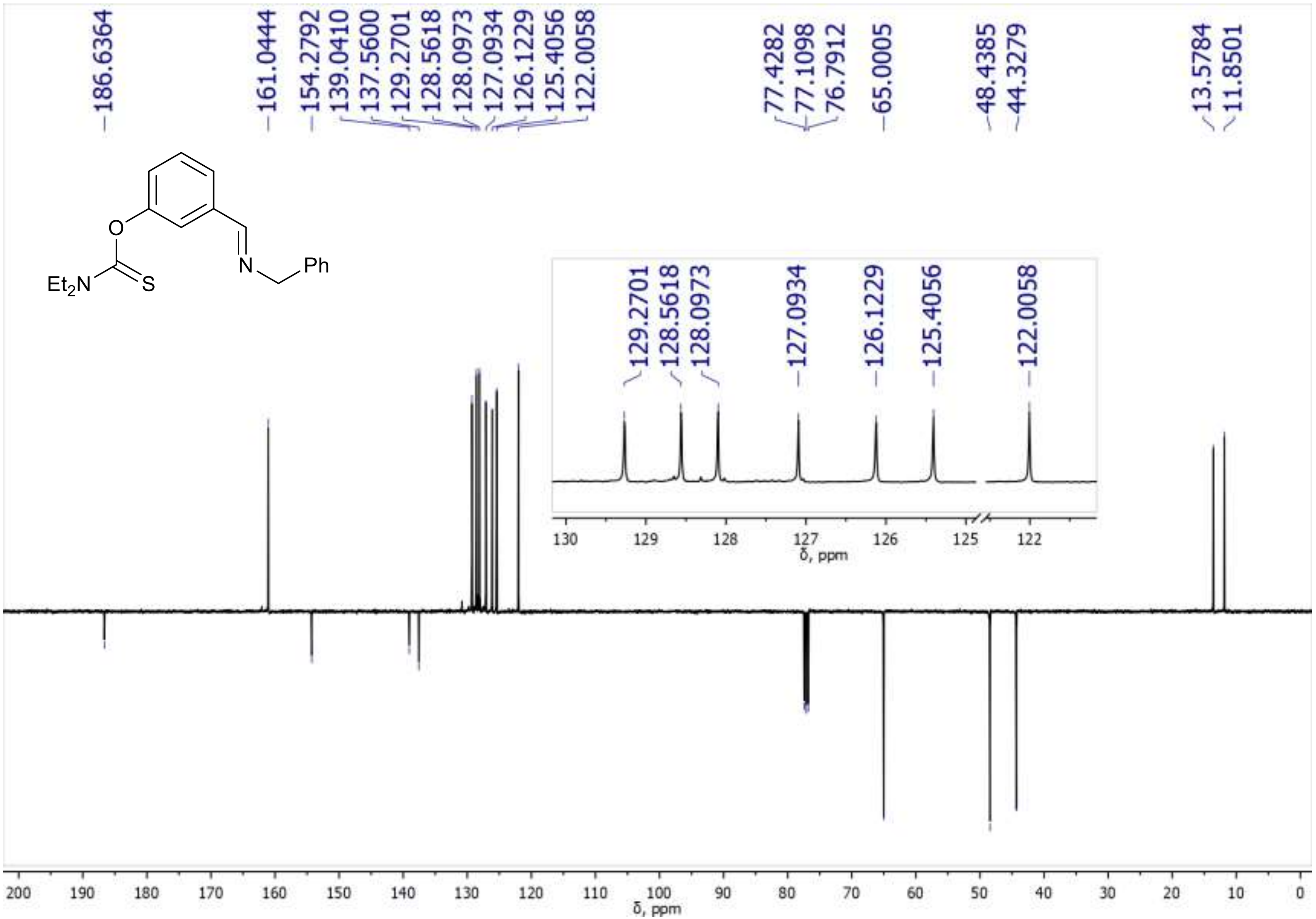

Figure S8. $^{13}C\{^{1}H\}$ NMR spectrum of ligand **5c** (100.61 MHz, $CDCl_3$)

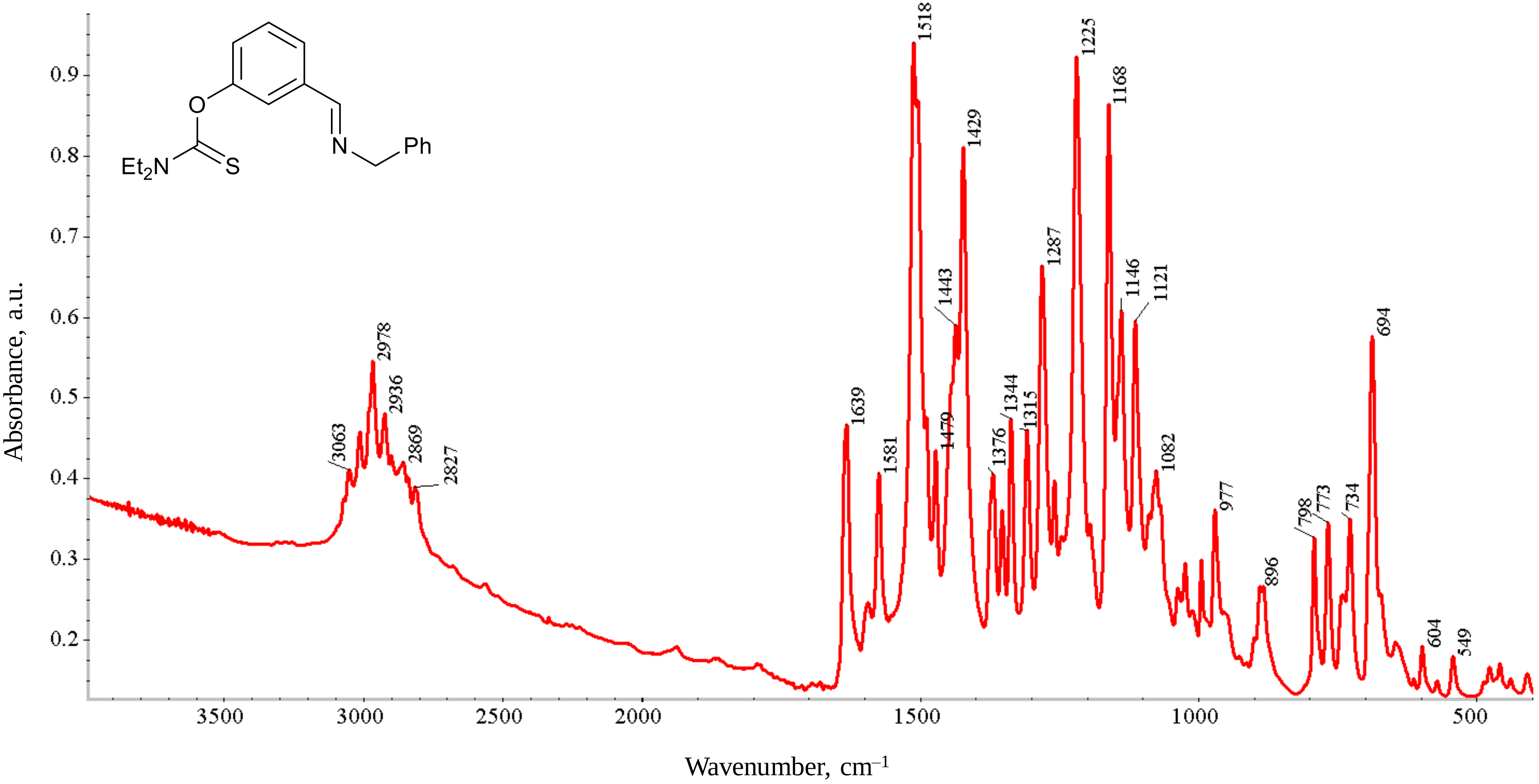

Figure S9. IR spectrum of ligand **5c**

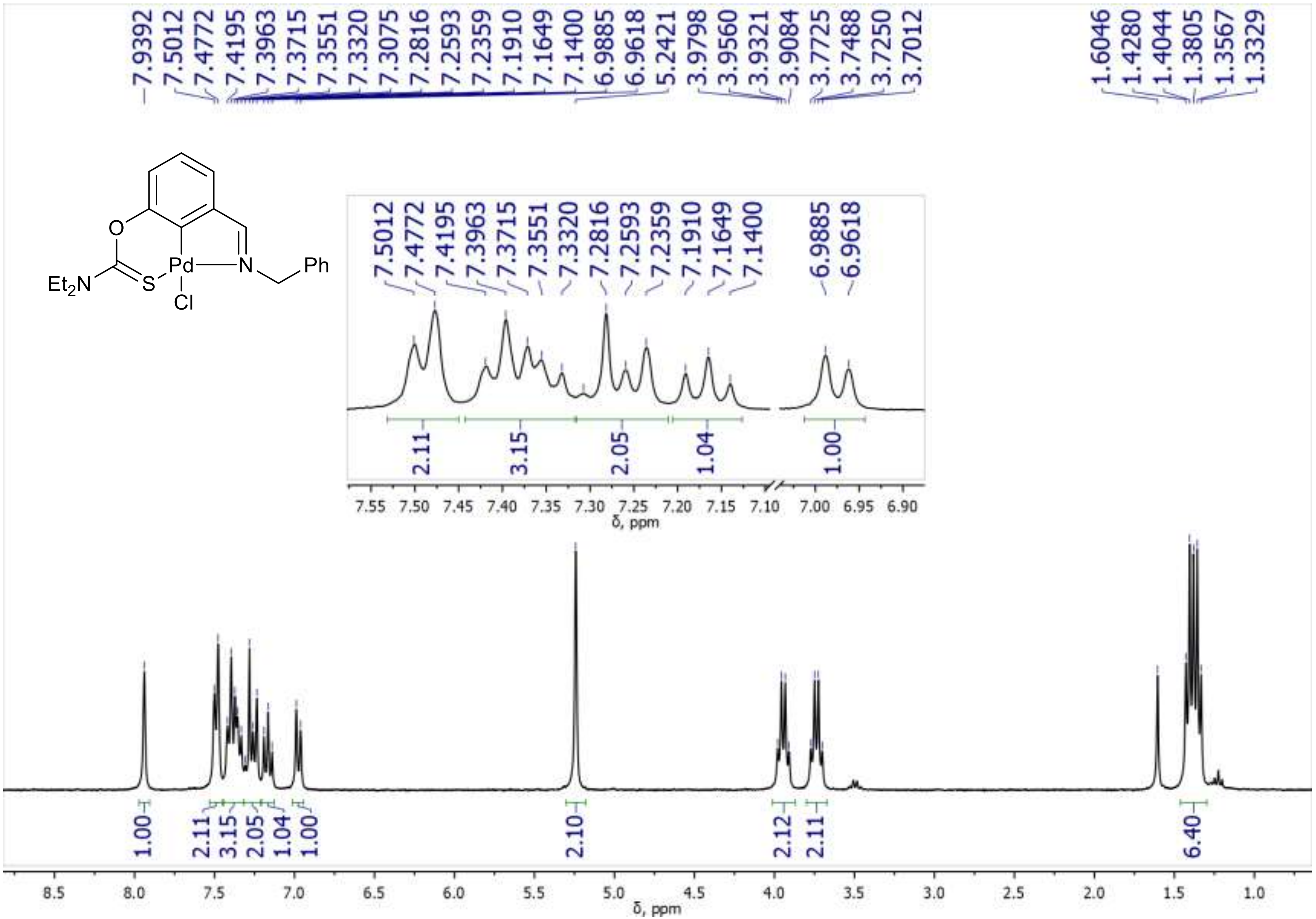

Figure S10. ^{1}H NMR spectrum of complex **8c** (300.13 MHz, $CDCl_3$)

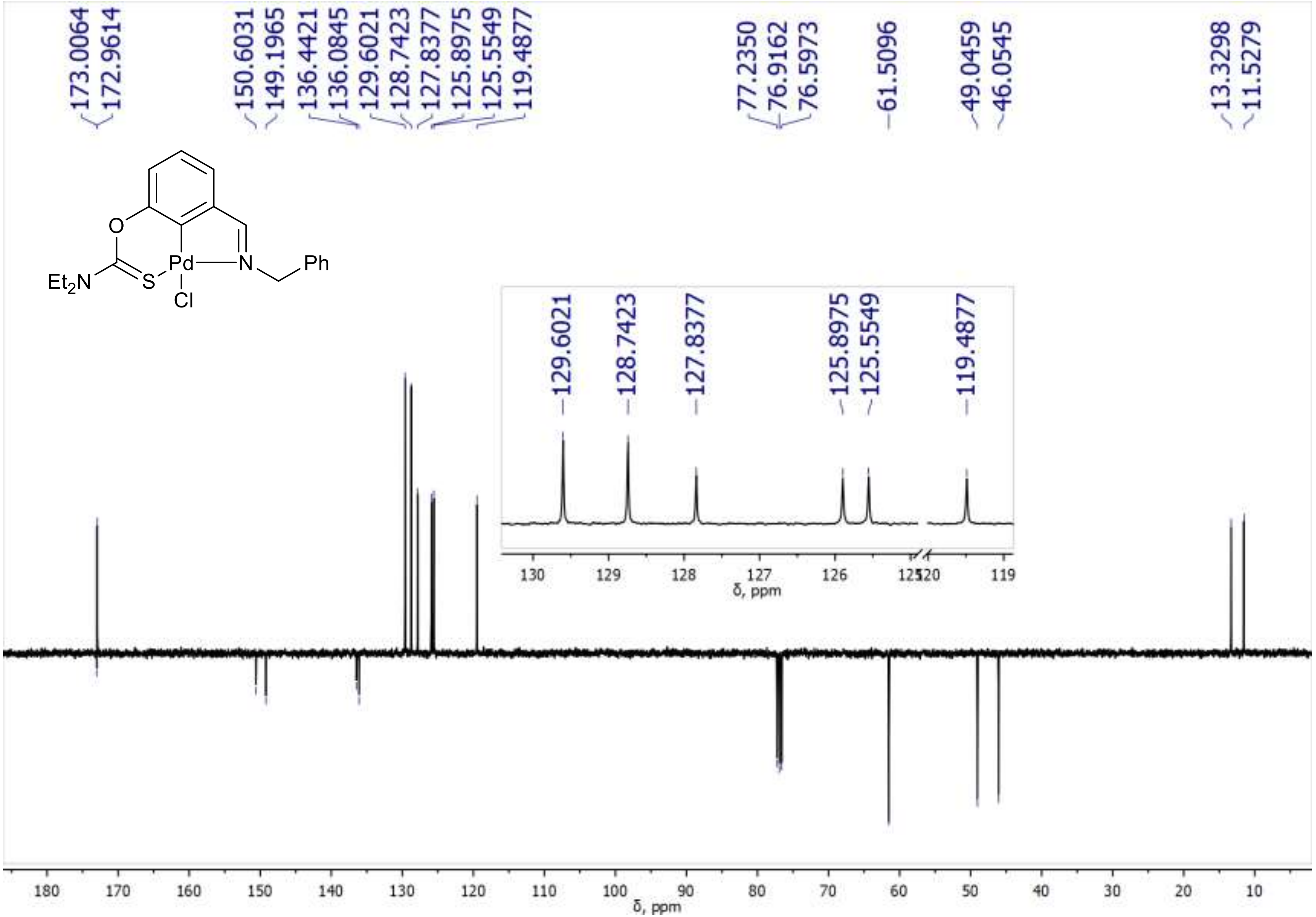

Figure S11. $^{13}C\{^1H\}$ NMR spectrum of complex **8c** (100.61 MHz, $CDCl_3$)

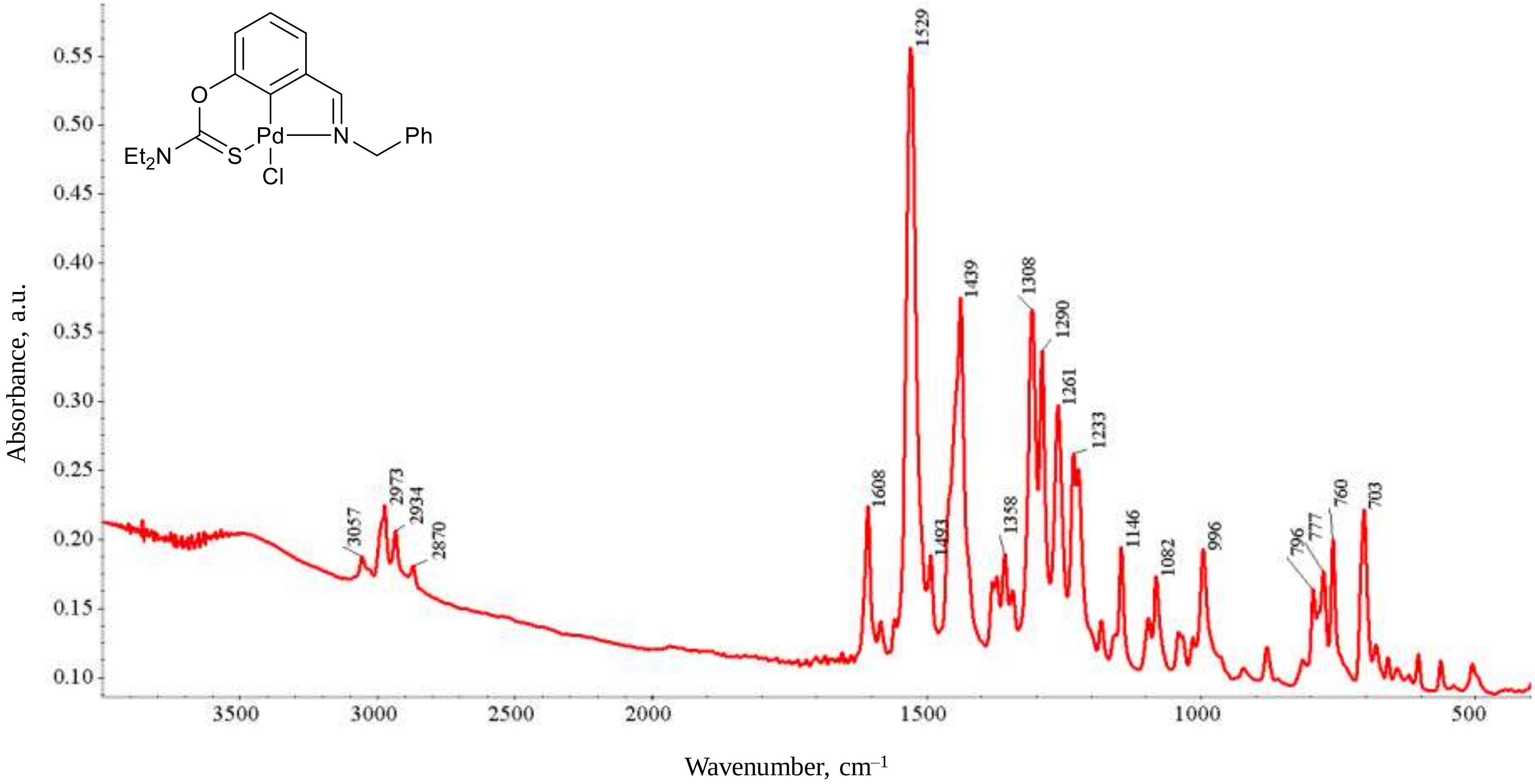

Figure S12. IR spectrum of complex **8c**

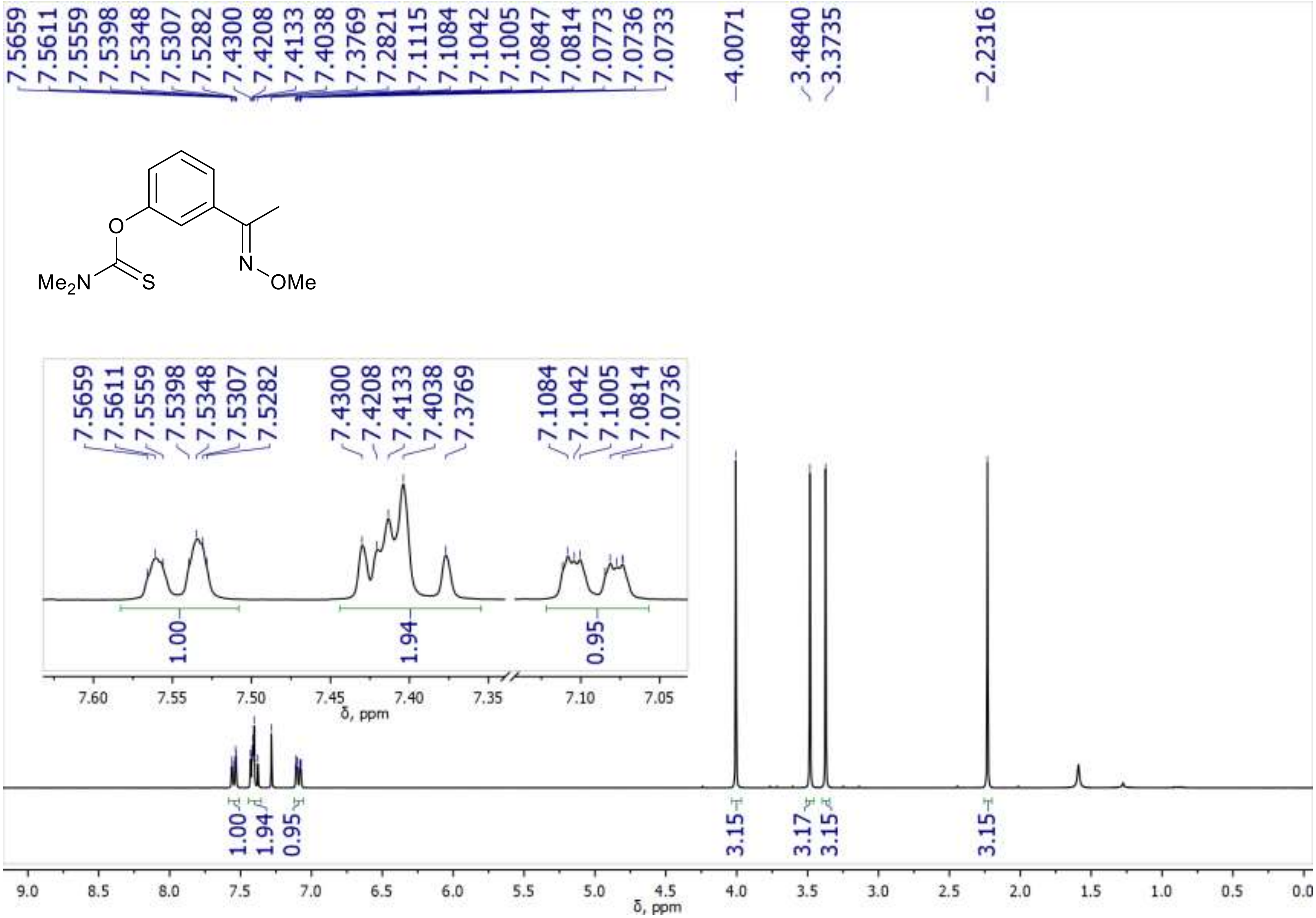

Figure S13. ^{1}H NMR spectrum of ligand **6** (300.13 MHz, $CDCl_3$)

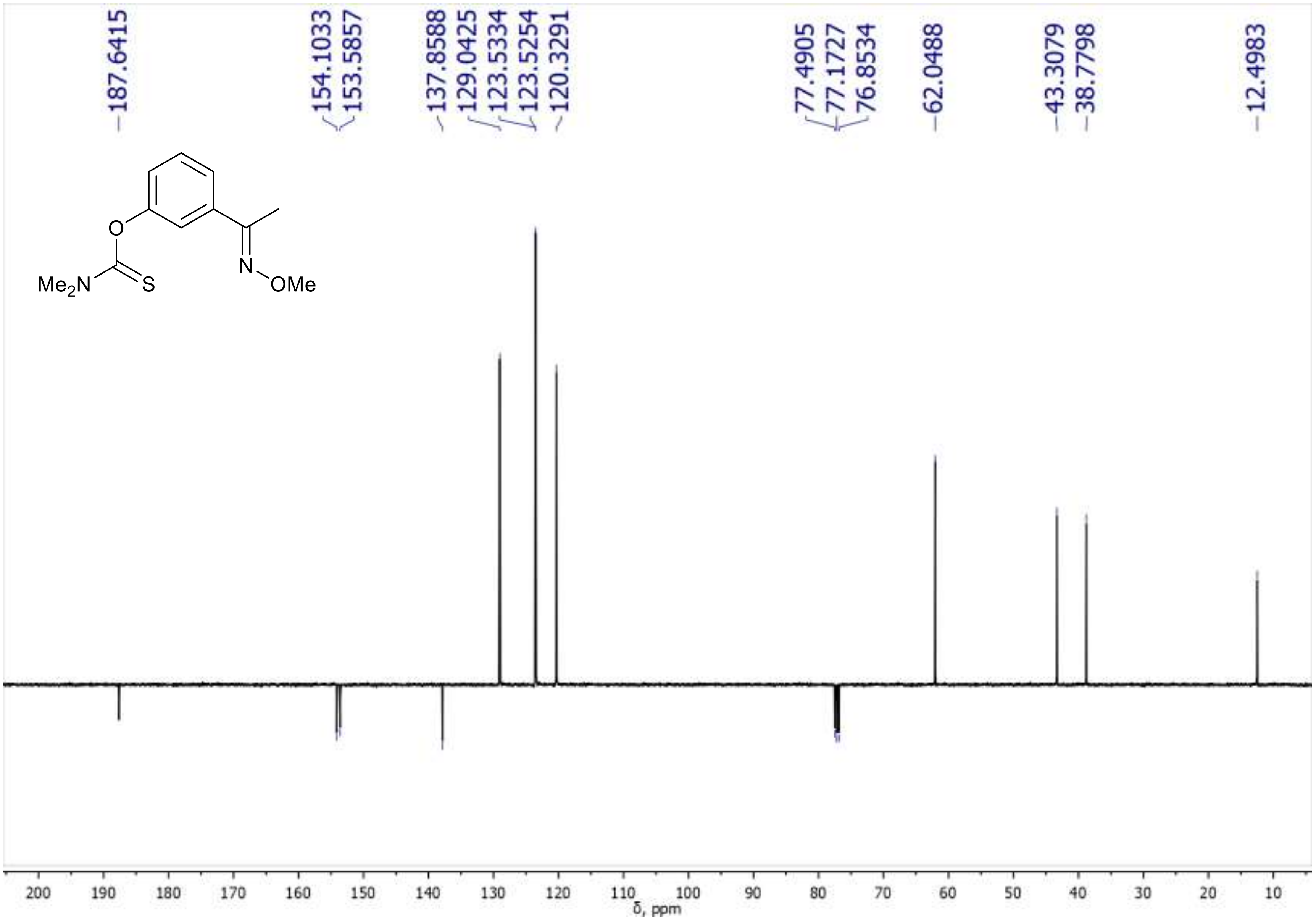

Figure S14. $^{13}C\{^1H\}$ NMR spectrum of ligand **6** (100.61 MHz, $CDCl_3$)

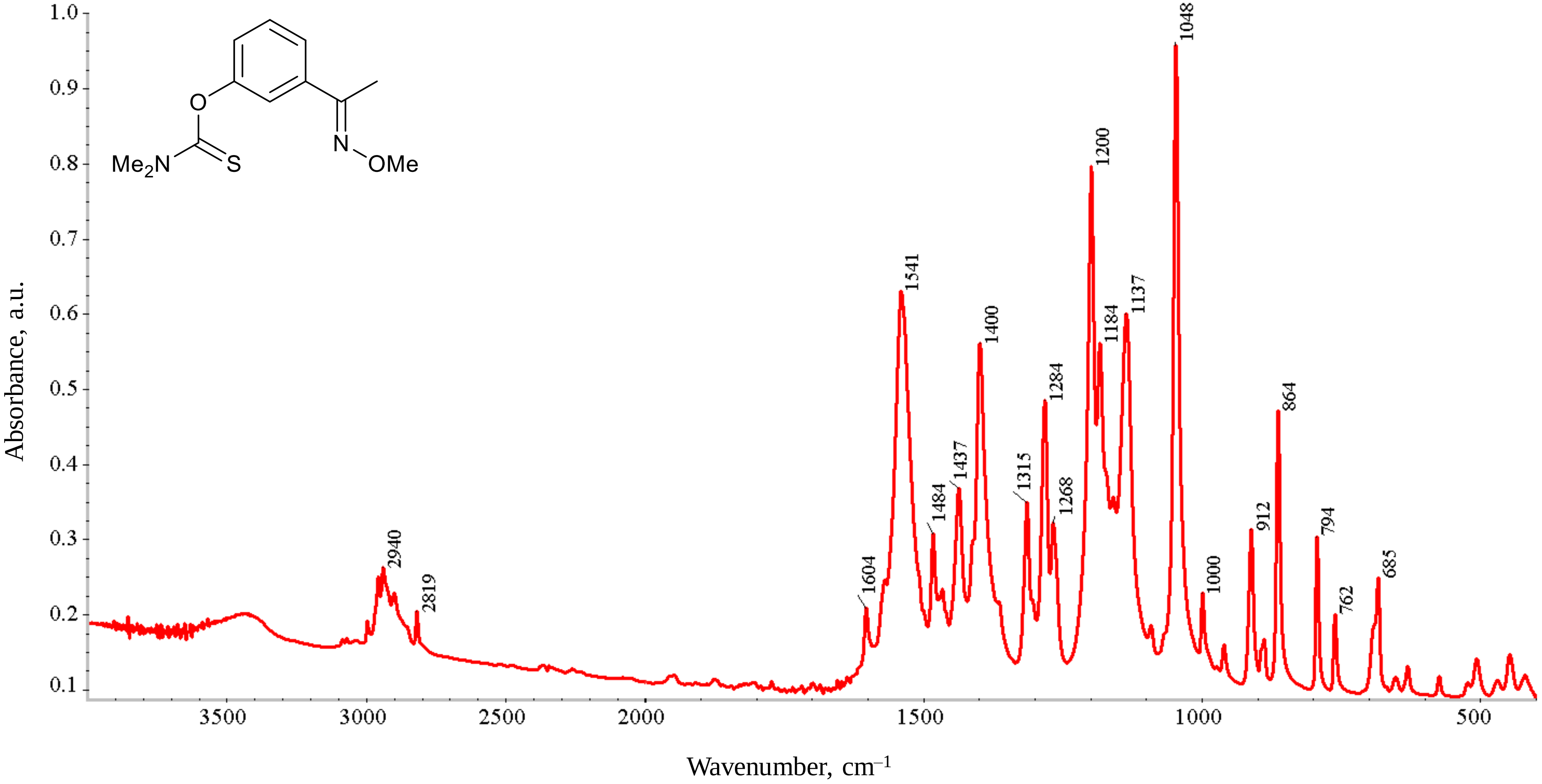

Figure S15. IR spectrum of ligand **6**

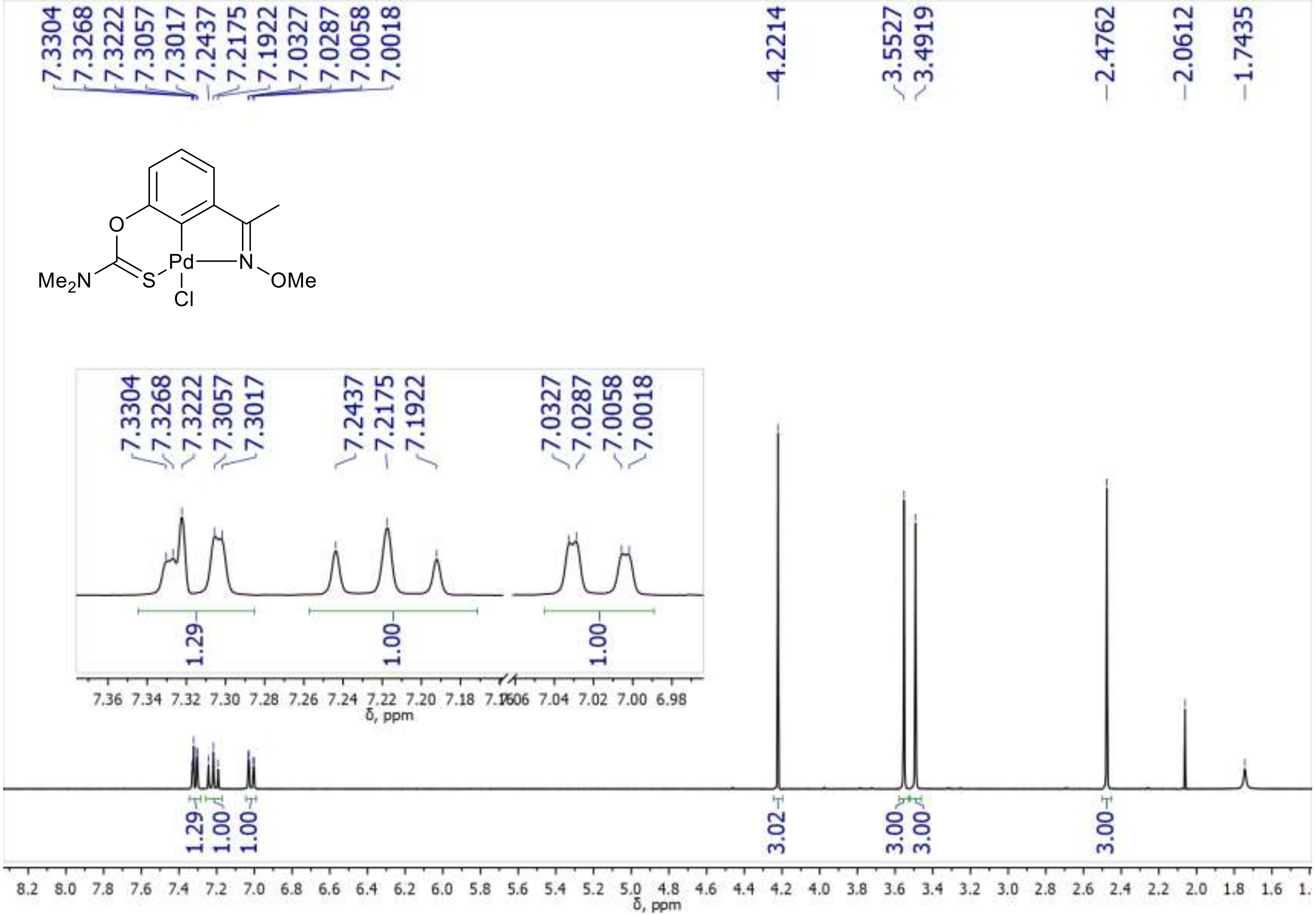

Figure S16. ^{1}H NMR spectrum of complex **9** (300.13 MHz, $CDCl_3$)

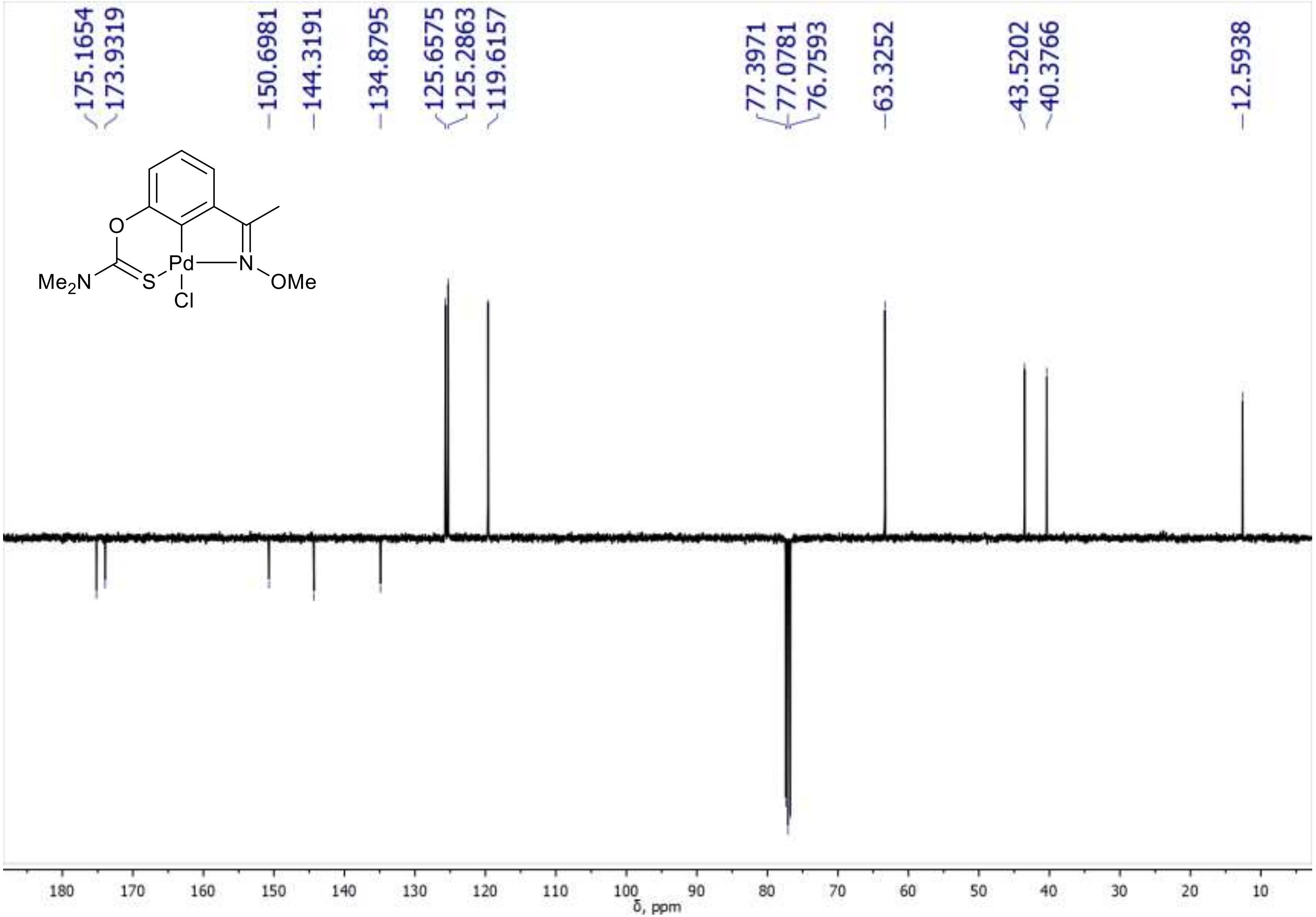

Figure S17. $^{13}C\{^{1}H\}$ NMR spectrum of complex **9** (100.61 MHz, $CDCl_3$)

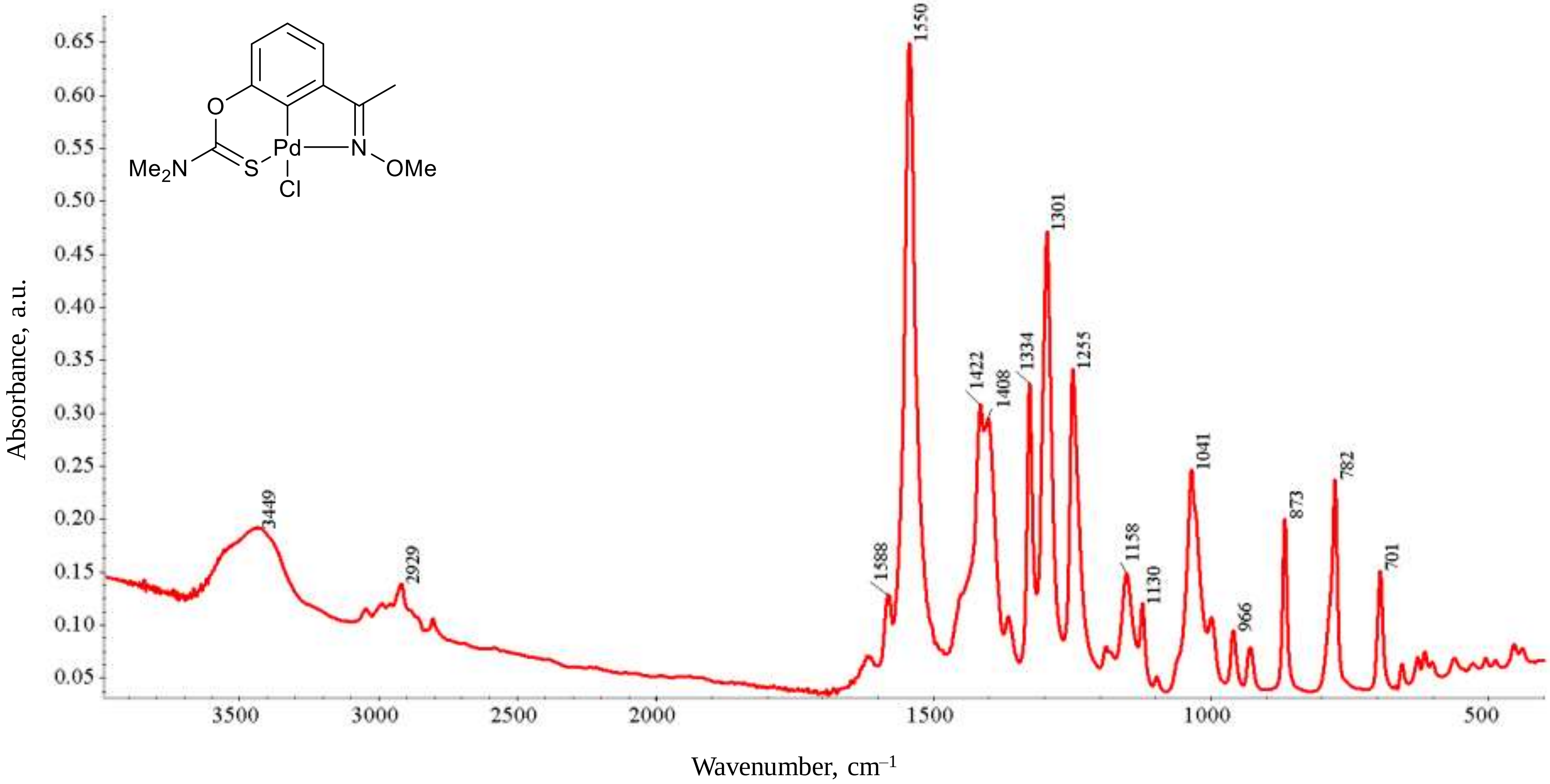

Figure S18. IR spectrum of complex **9**

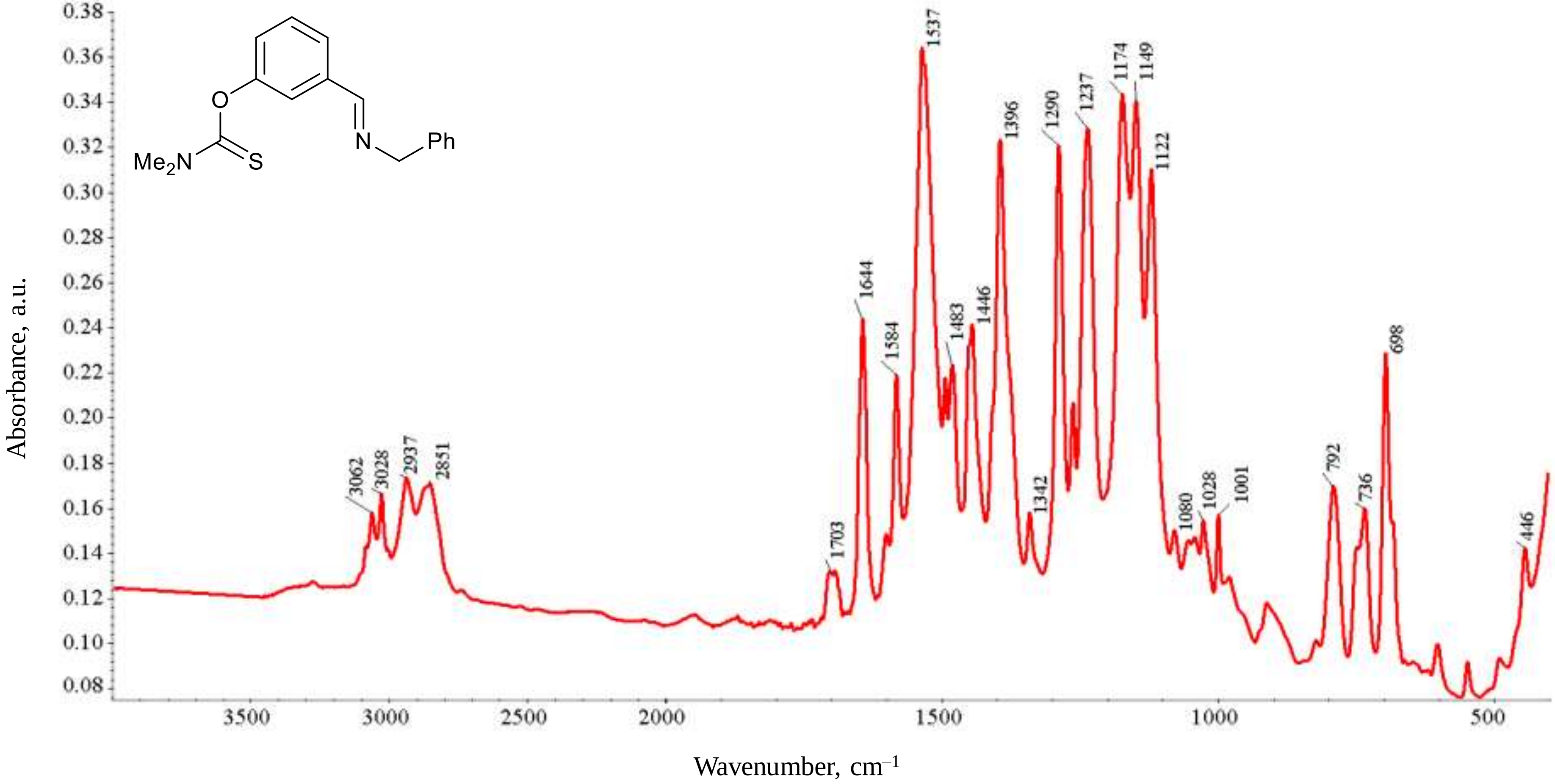

Figure S19. IR spectrum of ligand **4c**

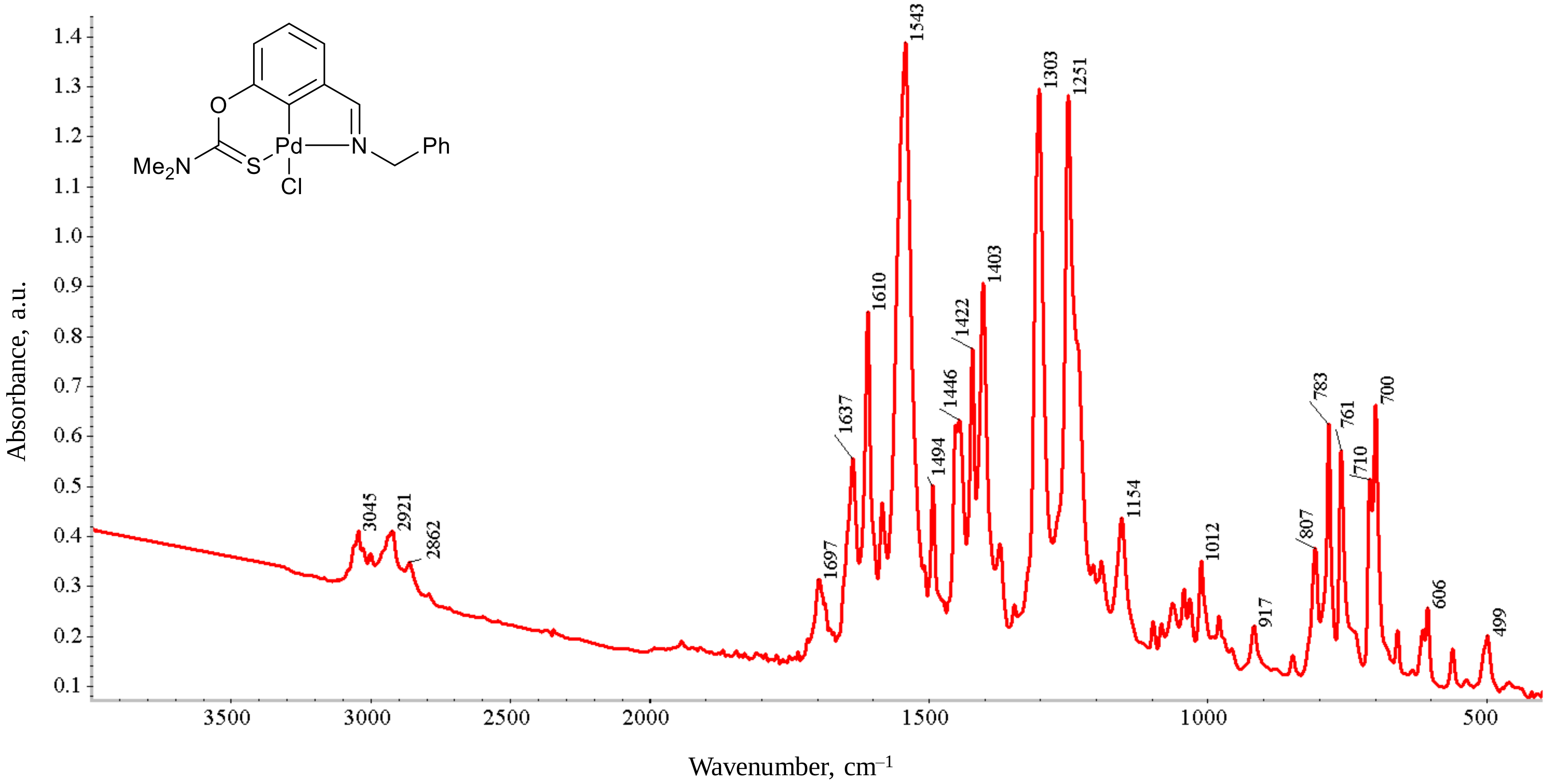

Figure S20. IR spectrum of complex **7c**

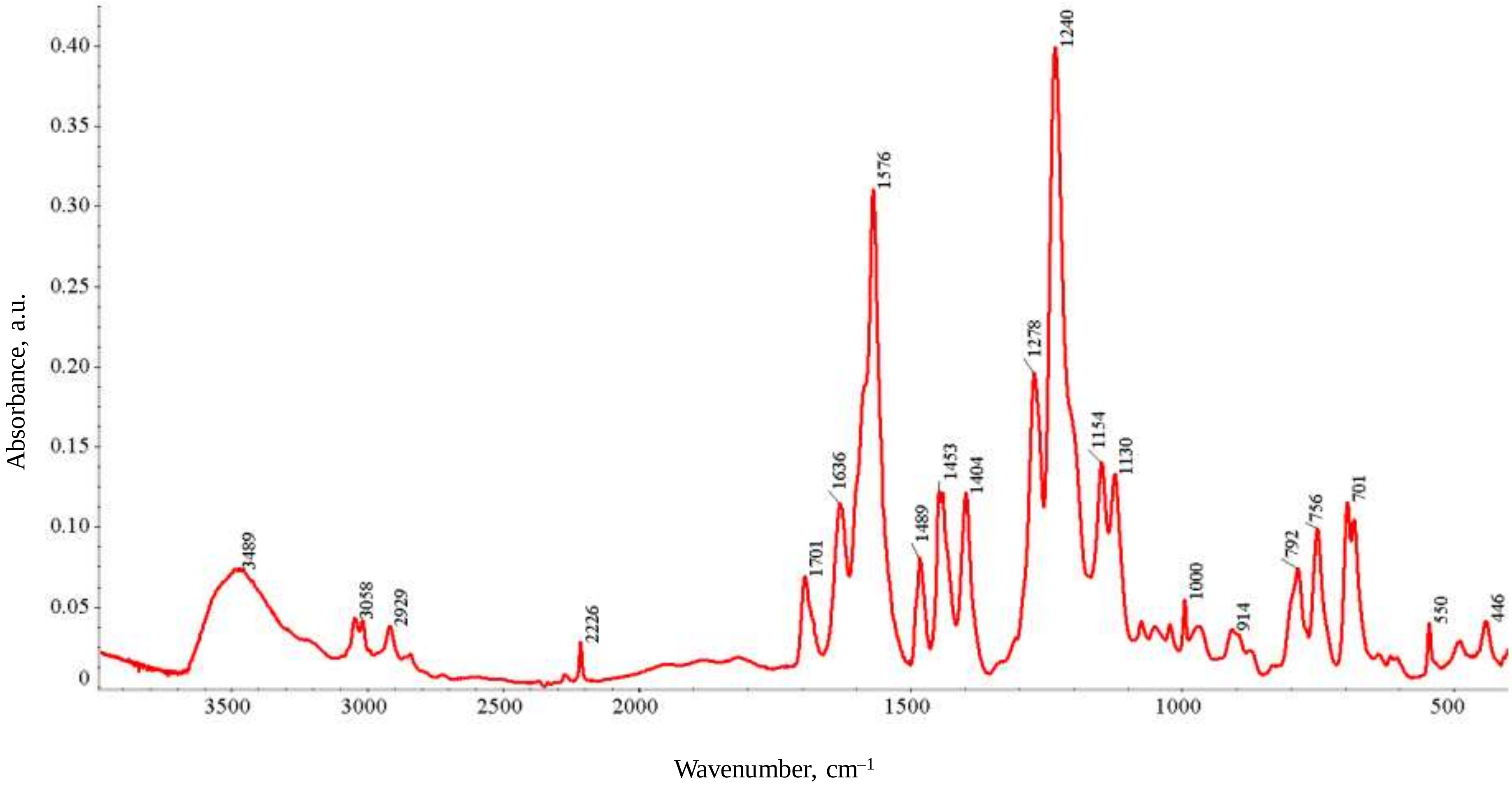

Figure S21. IR spectrum of the ground mixture of ligand **4c** and $PdCl_2(NCPh)_2$ (in 1 h after grinding)

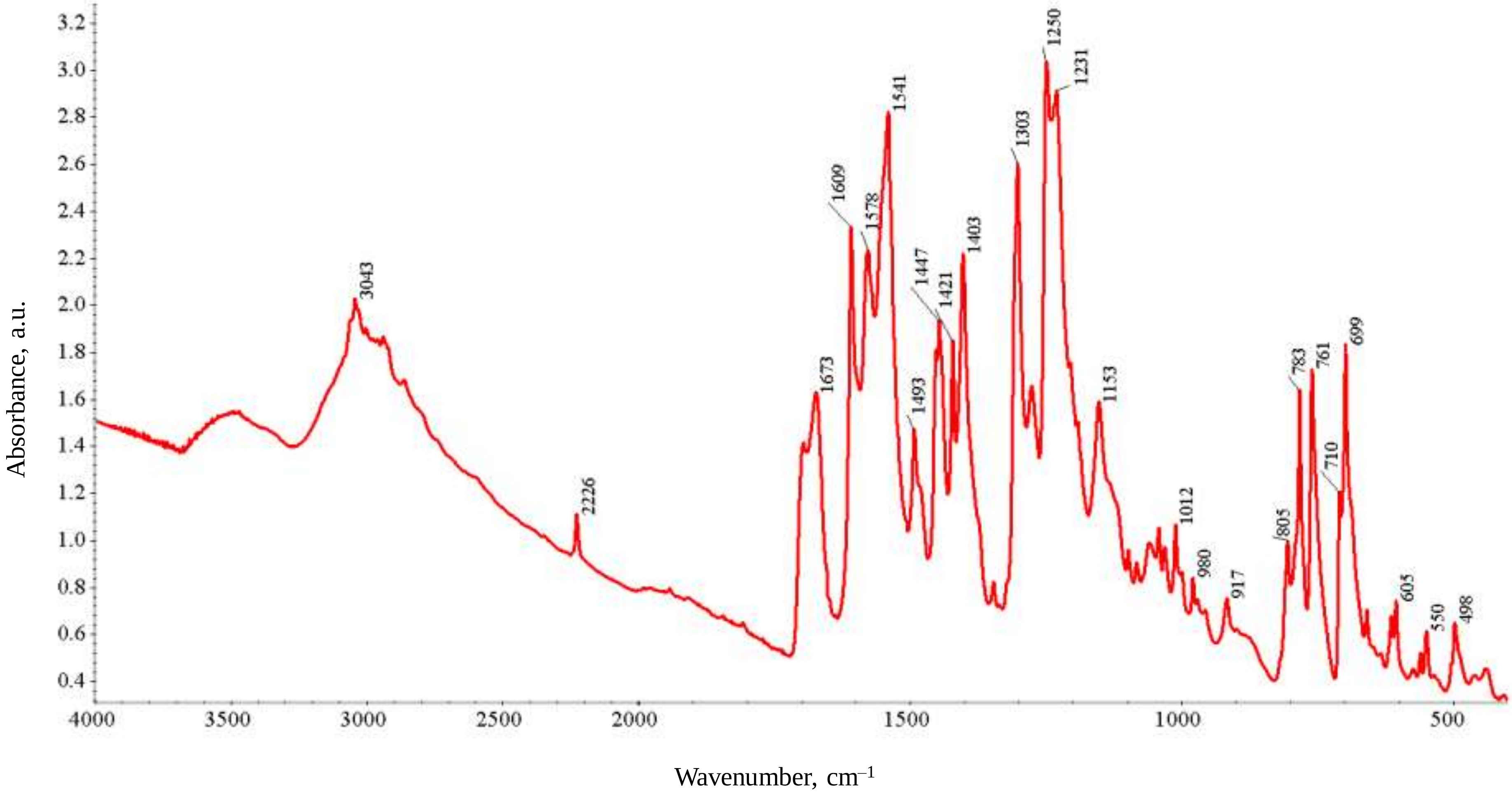

Figure S22. IR spectrum of the solid residue obtained after heating the ground mixture of ligand **4c** and $PdCl_2(NCPh)_2$ (130–135 °C, 10 min)

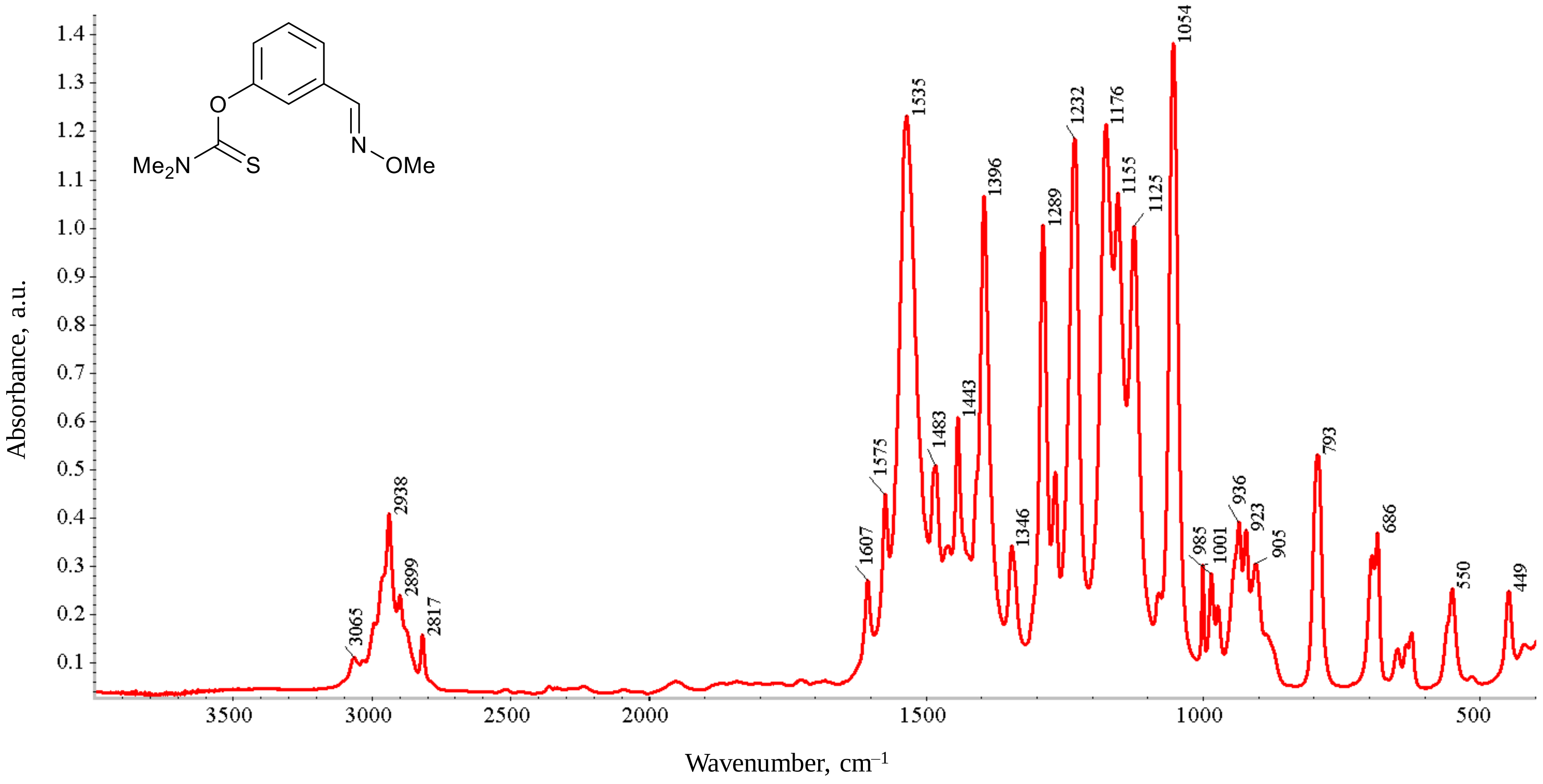

Figure S23. IR spectrum of ligand **4a**

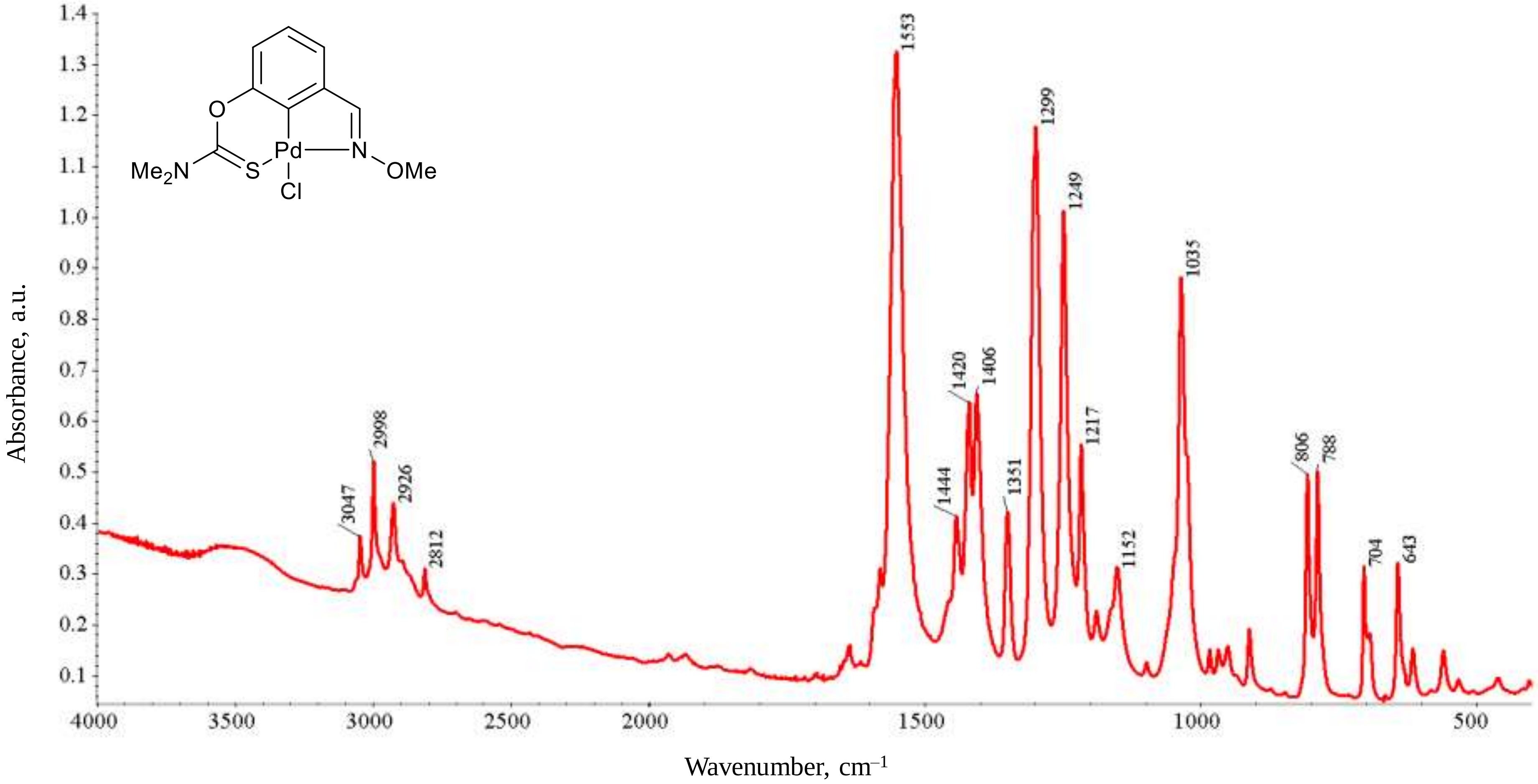

Figure S24. IR spectrum of complex **7a**

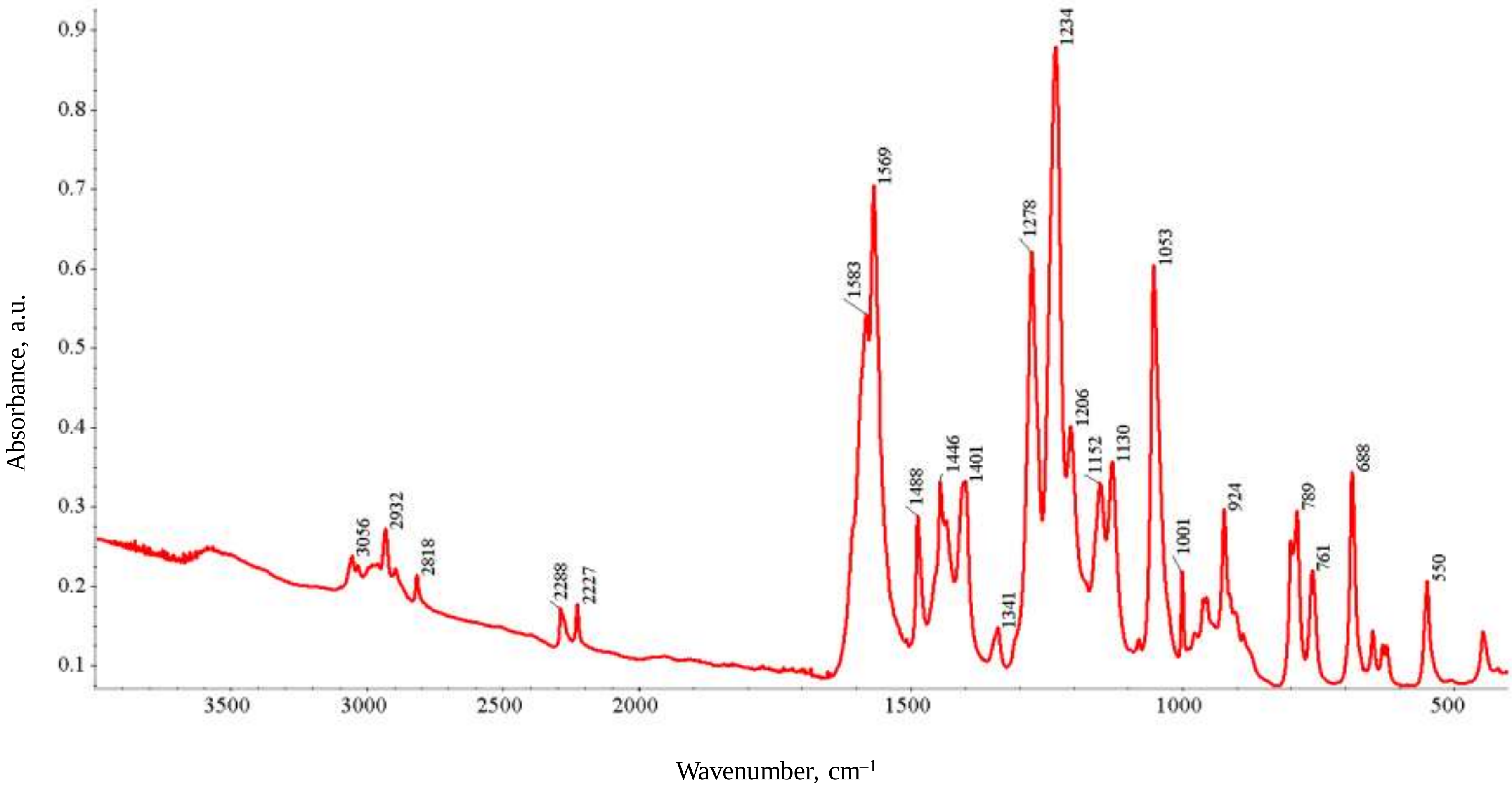

Figure S25. IR spectrum of the ground mixture of ligand **4a** and $PdCl_2(NCPh)_2$ (in 1.5 h after grinding)

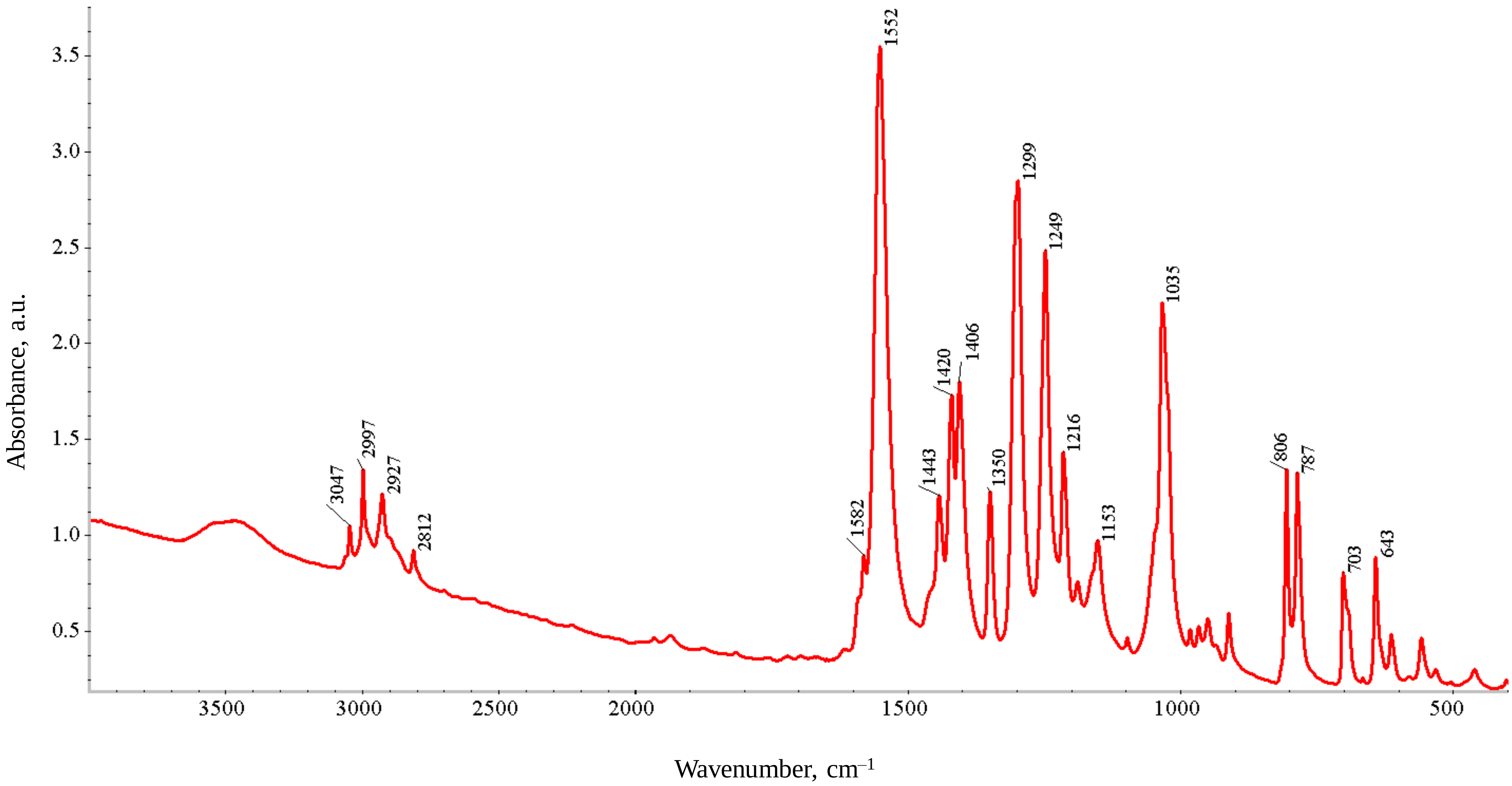

Figure S26. IR spectrum of the solid residue obtained after heating the ground mixture of ligand **4a** and $PdCl_2(NCPh)_2$ (105–107 °C, 15 min)

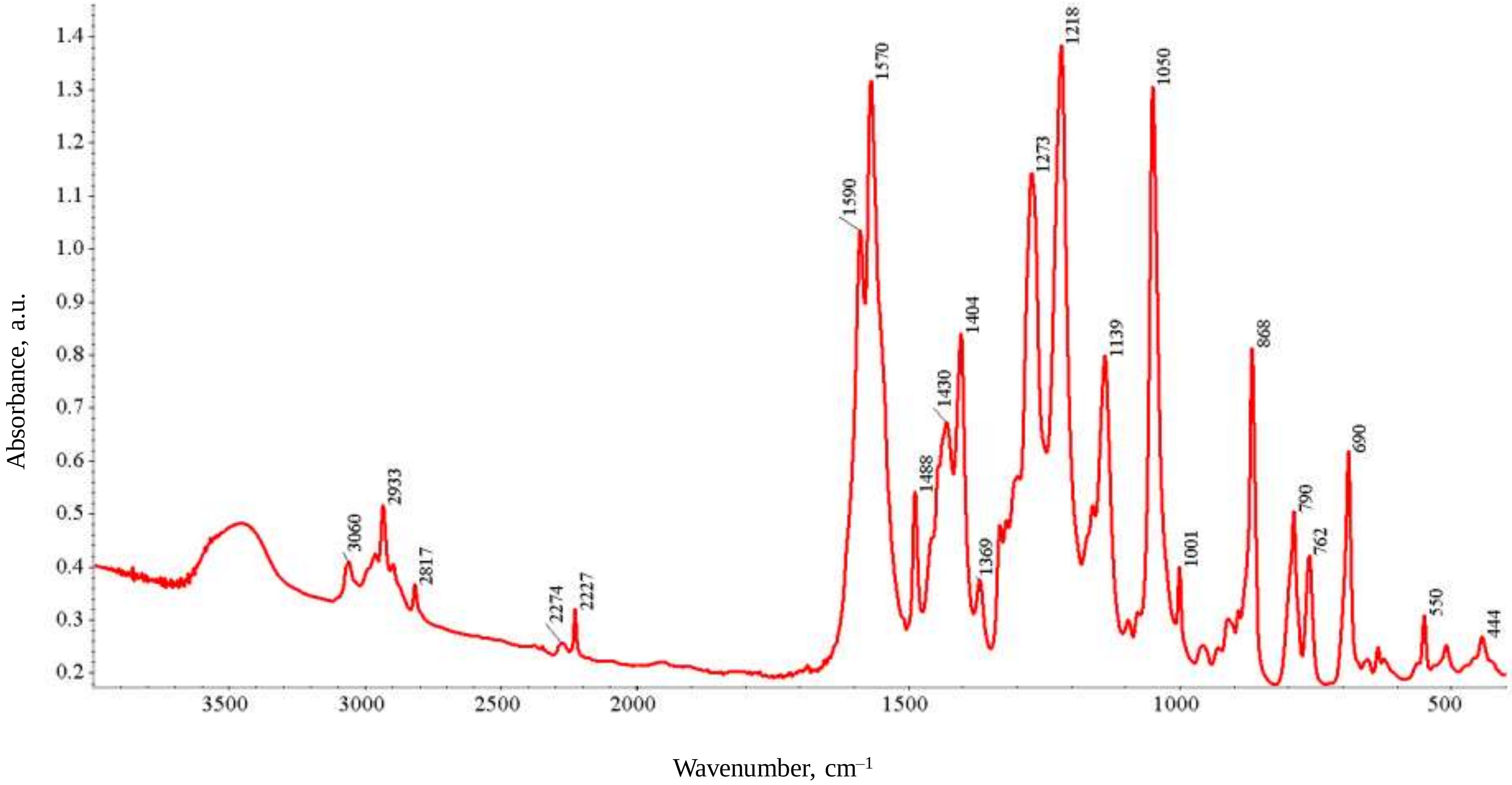

Figure S27. IR spectrum of the ground mixture of ligand **6** and $PdCl_2(NCPh)_2$ (in 1 h after grinding)

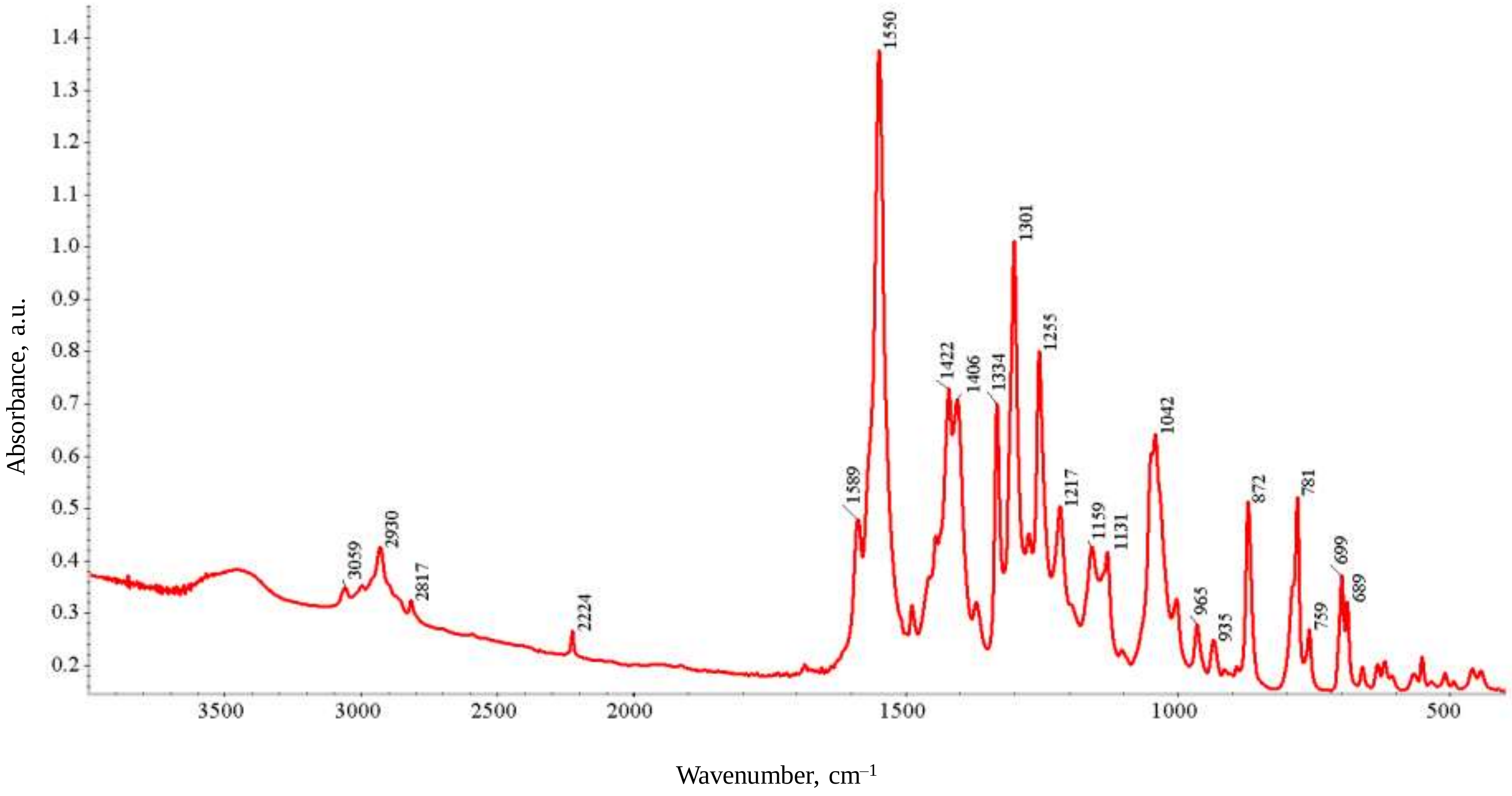

Figure S28. IR spectrum of the ground mixture of ligand **6** and $PdCl_2(NCPh)_2$ (in 1 day after grinding)

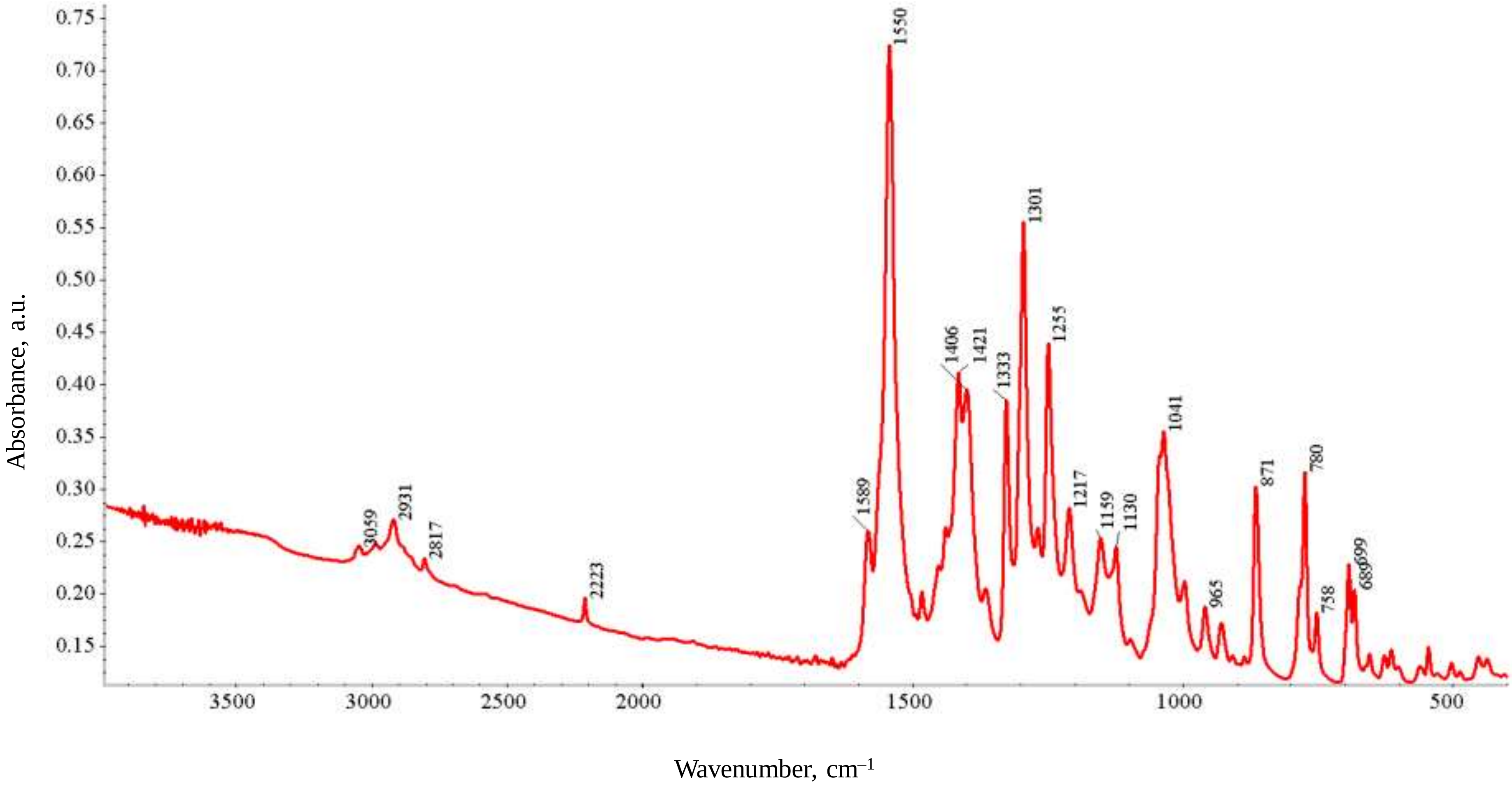

Figure S29. IR spectrum of the ground mixture of ligand **6** and $PdCl_2(NCPh)_2$ (in 2 days after grinding)

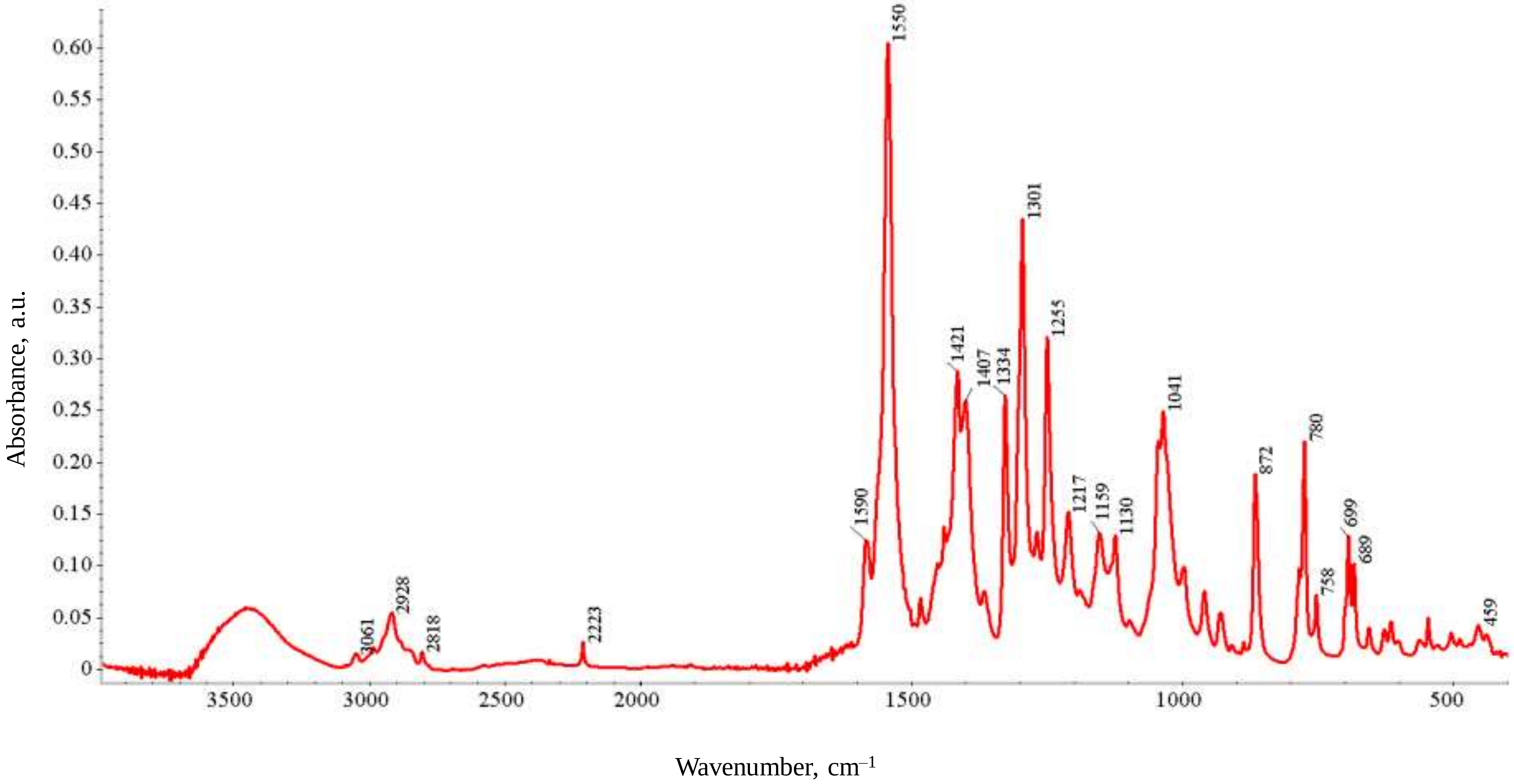

Figure S30. IR spectrum of the ground mixture of ligand **6** and $PdCl_2(NCPh)_2$ (in 1 week after grinding)

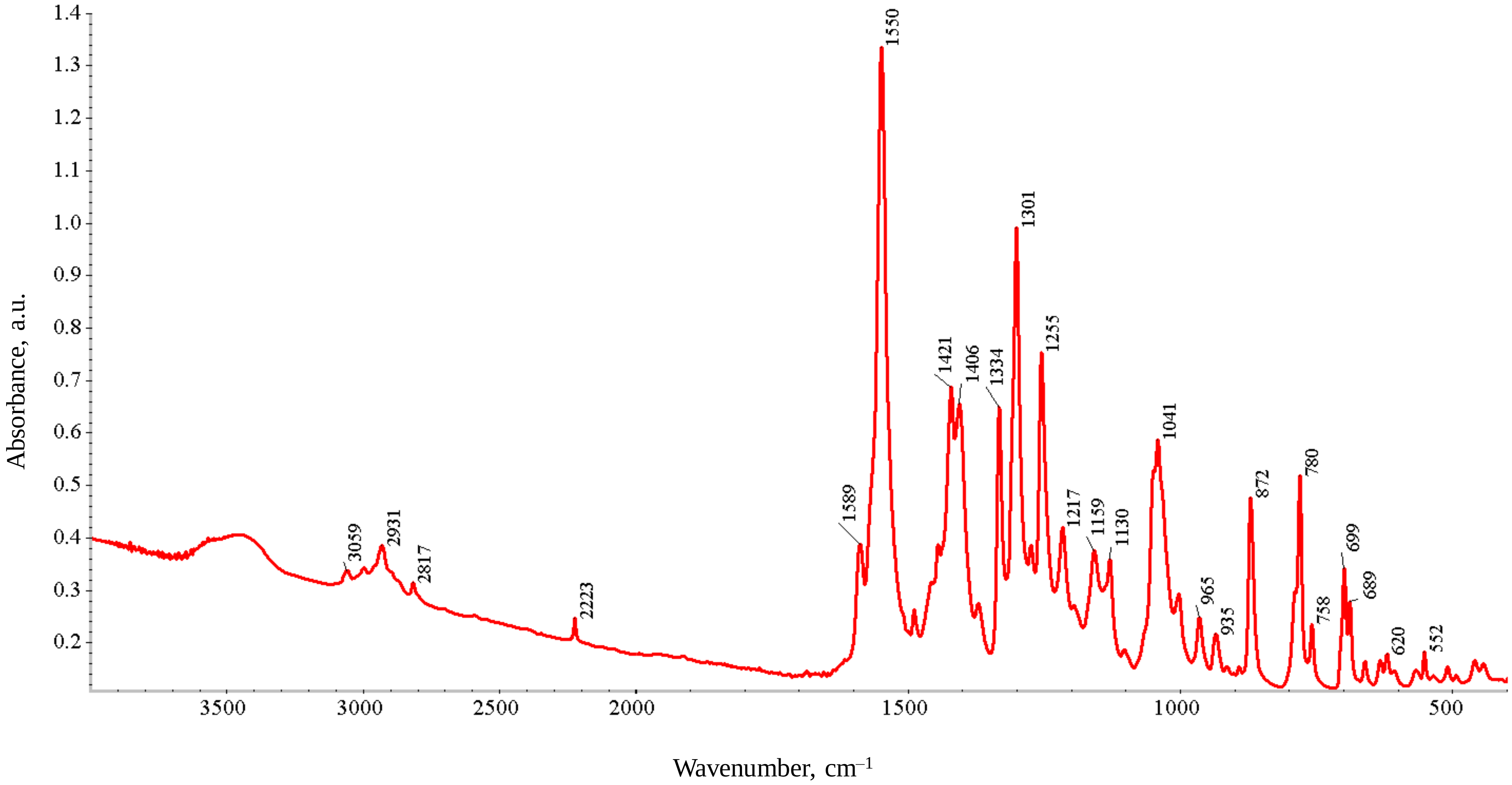

Figure S31. IR spectrum of the ground mixture of ligand **6** and $PdCl_2(NCPh)_2$ (in 2 weeks after grinding)

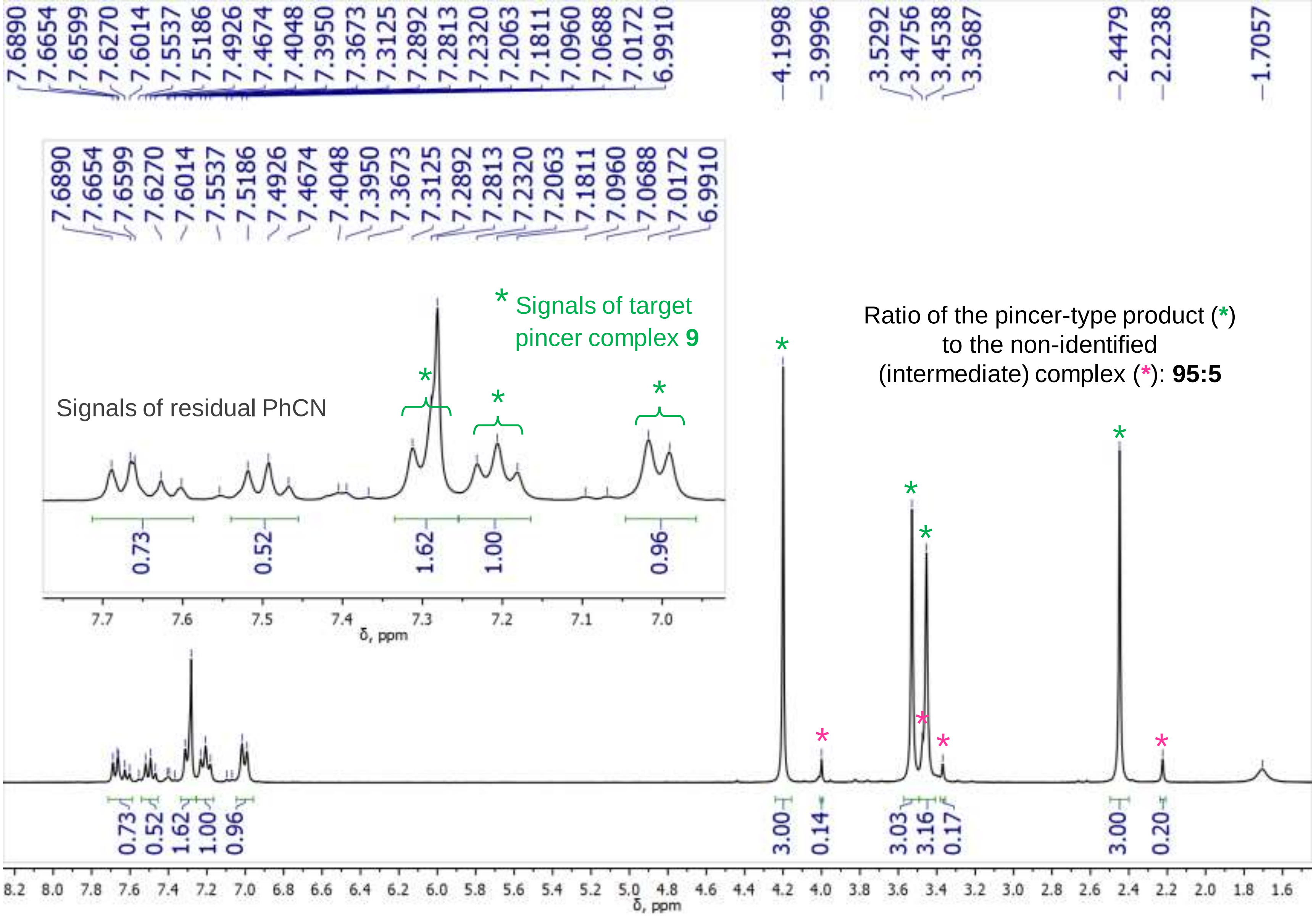

Figure S32. ^{1}H NMR spectrum of the free-flowing powder obtained in 2 weeks after grinding ligand **6** and $PdCl_2(NCPh)_2$, registered directly after dissolution in $CDCl_3$ (300 mHz)
